# A new survivin tracer tracks, delocalizes and captures endogenous survivin at different subcellular locations and in distinct organelles

**DOI:** 10.1038/srep31177

**Published:** 2016-08-12

**Authors:** Els Beghein, Isabel Van Audenhove, Olivier Zwaenepoel, Adriaan Verhelle, Ariane De Ganck, Jan Gettemans

**Affiliations:** 1 Department of Biochemistry, Faculty of Medicine and Health Sciences, Campus Rommelaere, A. Baertsoenkaai 3, Ghent University, Ghent, Belgium

## Abstract

Survivin, the smallest member of the inhibitor of apoptosis protein family, plays a central role during mitosis and exerts a cytoprotective function. Survivin is highly expressed in most cancer types and contributes to multiple facets of carcinogenesis. The molecular mechanisms underlying its highly diverse functions need to be extensively explored, which is crucial for rational design of future personalized therapeutics. In this study, we have generated an alpaca survivin nanobody (SVVNb8) that binds with low nanomolar affinity to its target. When expressed as an intrabody in HeLa cells, SVVNb8 faithfully tracks survivin during different phases of mitosis without interfering with survivin function. Furthermore, coupling SVVNb8 with a subcellular delocalization tag efficiently redirects endogenous survivin towards the nucleus, the cytoplasm, peroxisomes and even to the intermembrane space of mitochondria where it presumably interacts with resident mitochondrial survivin. Based on our findings, we believe that SVVNb8 is an excellent instrument to further elucidate survivin biology and topography, and can serve as a model system to investigate mitochondrial and peroxisomal (survivin) protein import.

Survivin (SVV; also known as BIRC5; 16.5 kDa) is the smallest member of the Inhibitor of Apoptosis Protein (IAP) family. The protein comprises 142 amino acids organized in two domains: an N-terminal baculovirus-IAP repeat (BIR) domain, linked to a C-terminal α-helix^1^. X-ray crystallography shows that the protein forms a dimer in solution^2^, but both monomeric as dimeric SVV appear *in vivo*^3^. Although SVV biology is still complex, its bifunctional role in mitosis and apoptosis are well-recognized.

SVV is of key importance during different stages of mitosis. This is reflected by (I) expression of the protein, which is cell cycle-dependent and peaks at G2/M^4^ and (II) the pleiotropic mitotic defects caused by SVV interference^5^,^6^,^7^. SVV is essential for chromosome alignment, sister chromatid segregation and cytokinesis^5^,^6^,^7^. Furthermore, SVV plays a central role during metaphase-anaphase transition, where the protein is required for sustained checkpoint activation in response to lack of microtubule tension or, in other words, when syntelic or merotelic attachments arise at metaphase^5^,^6^. SVV operates from two distinct subcellular pools in order to perform its different functions during mitosis. On the one hand, SVV binds mitotic spindle microtubules during metaphase, anaphase and late telophase^4^. Thereby, it suppresses microtubule dynamics^3^,^8^ and reduces centrosomal microtubule nucleation^8^. On the other hand, SVV forms a subunit of the chromosomal passenger complex (CPC), which is of vital importance for correct chromosome segregation and cytokinesis^9^,^10^.

Mitochondrial SVV, but not cytosolic, exerts a cytoprotective effect^11^. Apoptotic stimuli induce rapid release of mitochondrial SVV in the cytosol, where it inhibits apoptosis by preventing activation or activity of initiator caspase-9 (caspase-dependent)^3^,^11^,^12^,^13^,^14^,^15^ or by restraining apoptosis-inducing factor in the mitochondrial intermembrane space (caspase-independent)^3^,^15^.

SVV is highly expressed in most cancer types, compared to untransformed tissues^1^,^16^. Moreover, SVV expression in tumour cells is mainly cell cycle-independent, which indicates an increased anti-apoptotic role of SVV^17^. This theory is further strengthened by the fact that only tumour cells harbour a cytoprotective mitochondrial pool of SVV. This pool is selectively expanded in response to cellular stress. In this way, cancer cells elevate the anti-apoptotic threshold and adapt to environmental stress^11^. High SVV expression also enhances tumour cell migration and invasion^18^, stimulates metastasis^18^,^19^ and maintains cancer stem cell integrity^20^, which are other hallmarks of carcinogenesis. Consequently, high SVV expression indicates poor prognosis and high tumour recurrence^16^ and is associated with chemo- and radiotherapy resistance^21^,^22^.

SVV’s protuberant role in carcinogenesis and its differential expression in tumour versus untransformed tissue, instigated development of SVV-based cancer therapies^23^. Unfortunately, SVV biology remains extraordinarily complex and controversial^24^ and a better understanding of its biochemical pathways is vital for efficient design of personalized therapeutics^23^,^24^.

We and others have used single-domain antibodies or nanobodies (Nbs or VHHs; Variable domain of Heavy chain of Heavy chain antibodies) as bona fide research tools to investigate protein function and to unravel biochemical pathways^25^,^26^,^27^,^28^,^29^. The unique biochemical and biophysical properties of nanobodies and their potential of targeting novel epitopes, render them superior to antibodies or antibody-fragments^30^,^31^. Moreover, nanobodies represent a complementary strategy to RNAi, as they can be used to study functions of structural (‘undruggable’) proteins^32^. Here, we present a SVV nanobody that binds SVV with high affinity and accurately tracks SVV during different phases of mitosis as intrabody, without perturbing its function. Moreover, equipping the nanobody with an appropriate delocalization tag successfully shuttles endogenous SVV towards different cell organelles (nucleus, mitochondria and peroxisomes). Our data demonstrate that this SVV nanobody is an excellent tool to further unravel the complex SVV topography and biology.

## Results

### Characterization of survivin nanobodies

**Figure 1:**
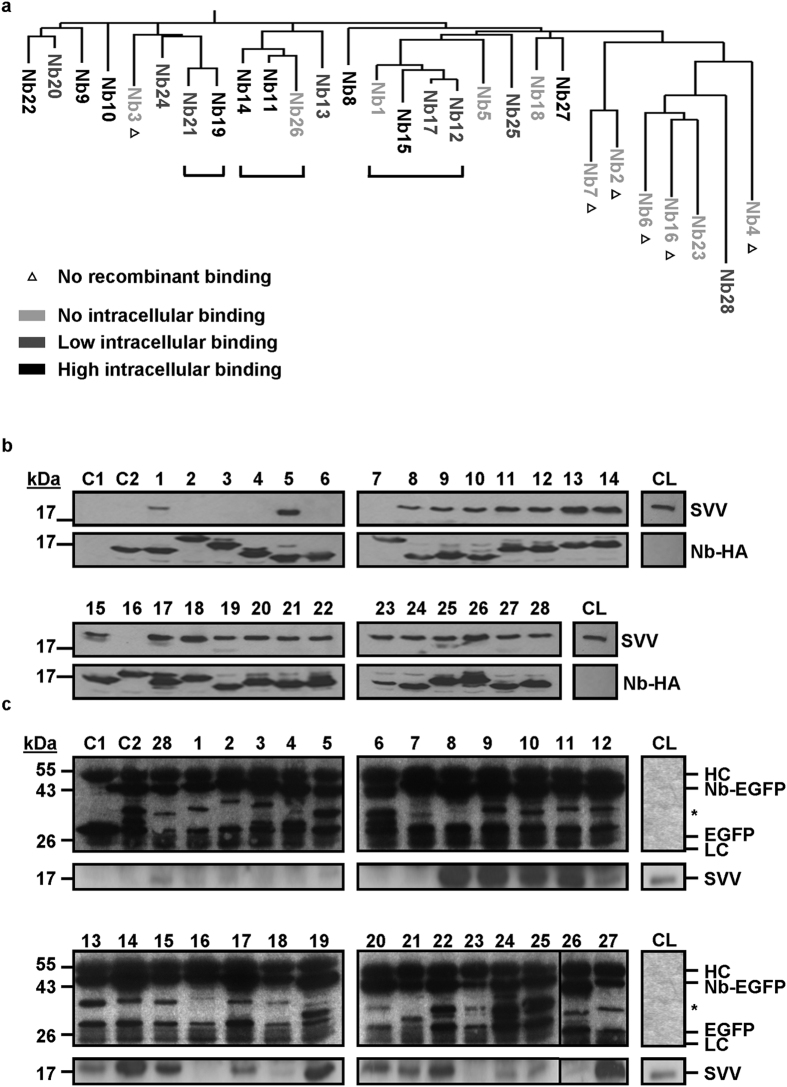
Validation and characterization of SVV nanobodies. (**a**) Phylogenetic tree of 28 SVV nanobodies representing their interrelations. The nanobodies can be classified into 22 different groups according to their amino acid sequence, of which 3 groups (depicted by horizontal solid lines) contain several members. Δ represents non-functional recombinant nanobodies. Unlabelled nanobodies bind SVV recombinantly. Nanobodies were additionally classified according to their intracellular binding capacity. This results in the following candidates for further use: Nb8-11, Nb14-15, Nb19, Nb22 and Nb27. (**b**) Pull-down experiment of endogenous SVV from HeLa cervix cancer cell lysate with recombinant HA-tagged nanobodies by means of anti-HA agarose beads. A negative control was included using HeLa cell lysate with anti-HA agarose beads only (C1). Cells expressing CapGNb2, a nanobody targeting the capping protein CapG, were used as a negative control (C2). Nanobodies were blotted with anti-HA antibody. CL = crude lysate. Full-length blots are presented in Supplementary Figure S4. (**c**) Immunoprecipitation experiment of endogenous SVV in HEK293T cells transfected with EGFP-tagged nanobodies, by means of an anti-GFP antibody. Two negative controls were included using cell lysates with EGFP only (C1) or CapGNb4-EGFP expression (C2). An anti-GFP antibody was used to visualize the nanobodies. LC = light chain, HC = heavy chain of the anti-GFP antibody. CL = crude lysate. *Depicts breakdown products of the EGFP-tagged nanobody construct. Full-length blots are presented in Supplementary Figure S4.

We identified 28 different nanobodies following phage display, belonging to 22 different groups according to their amino acid sequence. A phylogenetic tree confirms that nanobodies belonging to the same group (Nb21 and 19; Nb14, 11 and 26; Nb1, 15, 17 and 12) are derived from a common antecedent sequence and are thus clustered (Fig. 1a). To assess antigen binding, recombinant HA-tagged nanobodies were used to pull down endogenous survivin (SVV) from HeLa cells, which was successful for 22 out of the 28 nanobodies (Fig. 1b). All nanobodies were successfully expressed in the bacterial lysate. Nb2, 3, 4, 6, 7 and 16 were not able to bind endogenous SVV, although they were expressed to the same extent as positive, SVV-binding nanobodies. Of note, all non-binding nanobodies are clustered in the phylogenetic tree, except for Nb3 (Fig. 1a). Next, nanobodies were evaluated for their ability to function as intrabodies. To this end, transiently expressed EGFP-tagged nanobodies were assessed for their target binding in HEK293T cells by means of co-immunoprecipitation (Fig. 1c). All nanobodies were successfully expressed intracellularly and 17 out of the 28 nanobodies (Nb 8–15, 17, 19–22, 24-25, 27-28) were able to recognize endogenous SVV in the complex eukaryotic environment, although to a different extent. Figure 1a gives an overview of the expression and binding characteristics of the 28 SVV nanobodies. Recombinant nanobodies that do not bind SVV, also do not function as an intrabody. Notably, the branch at the far right of the phylogenetic tree only contains nanobodies that do not bind intracellularly (Fig. 1a), except for Nb28 (which binds SVV to a low extent). The following nanobodies were categorized as strong intracellular binders: Nb8-11, Nb14-15, Nb19, Nb22 and Nb27.

### SVVNb8 binds full-length SVV with high affinity

**Figure 2:**
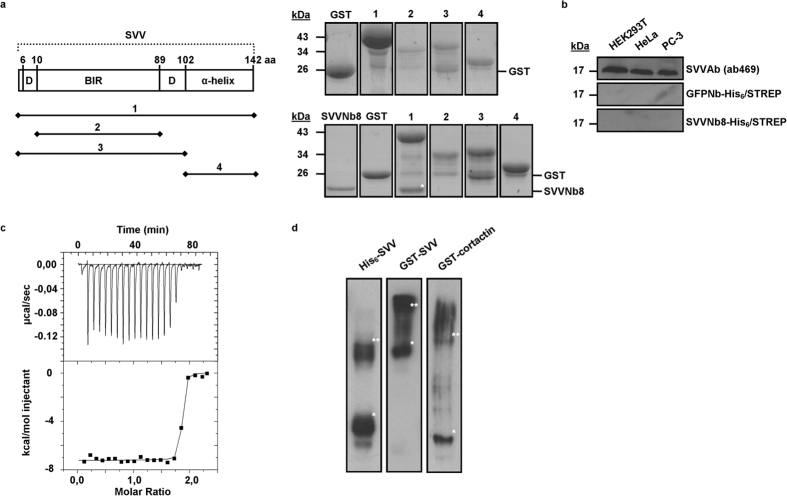
Detailed binding characterization of SVVNb8. (**a**) Epitope mapping experiment with GST only (control), GST-tagged full-length SVV (1), the BIR domain without (2) or with (3) dimer interfaces or the α-helix domain (4) as depicted at the left. The right upper panel shows the different recombinant GST fusion proteins. A Coomassie-stained gel of the GST-pull-down experiment is shown in the right lower panel. SVVNb8 (*) can only be found together with full-length SVV. (**b**) Representative blot on lysates of HEK293T, HeLa cervix cancer or PC-3 prostate cancer cells using recombinant His_6_/STREP-tagged SVVNb8 as Western blot reagent followed by an anti-His_6_ antibody. A commercial polyclonal anti-SVV antibody (ab469) and a His_6_/STREP-tagged GFP-targeting nanobody were used as positive and negative control, respectively. SVVNb8 does not detect SVV on Western blot. Full-length blots are presented in Supplementary Figure S4. (**c**) ITC profile of recombinant His_6_/STREP-tagged SVVNb8 (injected) with recombinant GST-SVV (sample cell). The upper panel shows raw data of heat release as a function of time, while the lower panel shows the fitted binding curve of total heat release per injection as a function of the molar ratio. SVVNb8 binds SVV with an affinity of K_d_ = 0.95 ± 0.53 nM and a molar ratio of 2:1 SVVNb8:SVV. (**d**) Western blot after native PAGE of recombinant purified His_6_-SVV, GST-SVV and GST-cortactin. GST-cortactin was used as a control and native proteins were detected with anti-SVV (left and middle) or anti-cortactin (right) antibody. Both His_6_-SVV and GST-SVV can form dimers in solution. *Depicts monomeric protein, **Depicts dimeric protein.

We performed a detailed biochemical characterization of survivin nanobody 8 (SVVNb8), which strongly binds SVV both *in vitro* and *in vivo*. The epitope to which SVVNb8 binds was determined by means of GST-pull-down experiments (Fig. 2a). SVV consists of two domains: an N-terminal BIR domain linked to a C-terminal α-helix. A dimer interface domain (D) precedes the BIR domain and separates it from the α-helix^24^. SVVNb8 only binds with full-length SVV, not the distinct SVV domains. This suggests that the nanobody recognizes (I) a linear epitope or conformational epitope which includes both domains or (II) a conformational epitope on one domain whose structure is lost when another domain is missing. As SVVNb8 could not detect denatured endogenous SVV on Western blot, we can confirm that the nanobody binds a conformational epitope (Fig. 2b). Subsequently, the binding affinity and stoichiometry of the SVVNb8 to GST-SVV binding were determined using isothermal titration calorimetry (Fig. 2c). A K_d_ value of 0.95 ± 0.53 nM and a molar ratio of 2 SVVNb8s to 1 GST-SVV could be derived. A reaction stoichiometry of 2:1 implies that GST-SVV forms a dimer in solution, which is confirmed by native PAGE (Fig. 2d). Dimerization is likely to be caused in part by the GST-tag and in part by SVV itself^2^, as cortactin (a true monomeric protein of 85 kDa^33^) also appears in two discrete bands when coupled to a GST-tag, as does His_6_-SVV (without GST-tag). In conclusion, SVVNb8 recognizes a conformational epitope and binds full-length SVV with high affinity (K_d_ = ~1 nM).

### A SVVNb8 intrabody accurately tracks SVV during different phases of mitosis

**Figure 3:**
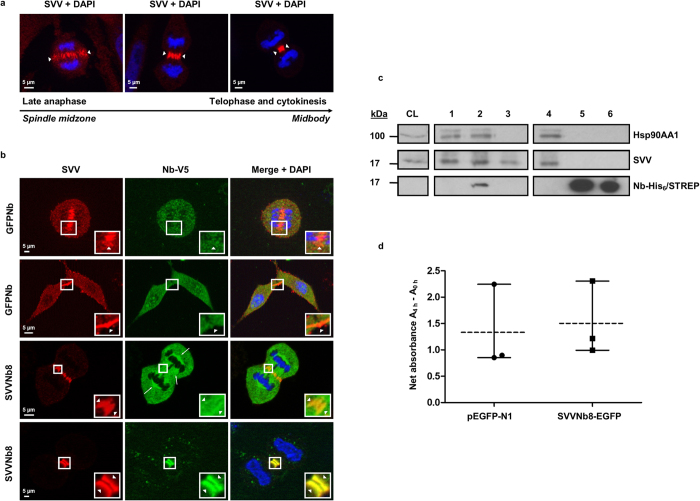
SVVNb8 colocalizes with SVV in different cell cycle stages without affecting cell viability. (**a**) Endogenous SVV is typically enriched at the spindle midzone and the midbody between anaphase and cytokinesis, as visualized in HeLa cervix cancer cells (arrowheads Δ). (**b**) HeLa cervix cancer cells transfected with a GFP-targeting nanobody (control) show no enrichment at the spindle midzone (first panel) or midbody (second panel). On the other hand, SVVNb8 is enriched at the spindle midzone (third panel) or midbody (lower panel) upon expression (boxed areas, arrowheads Δ). Boxed areas are enlarged in the insets. SVVNb8 is also enriched between sister chromatids and at the cell poles (arrows **→**) during late anaphase. Nuclei were visualized by means of DAPI and nanobodies with anti-V5 antibody. (**c**) Representative co-immunoprecipitation experiment of endogenous Hsp90AA1-SVV complex in MDA-MB-231 breast cancer cells by means of anti-SVV antibody (D-8) coupled on protein G sepharose beads. A negative control was included using cell lysate with protein sepharose beads only (lane 3), representing the level of aspecific SVV binding on naked beads. Crude lysates were incubated with GFPNb (lane 1) or SVVNb8 (lane 2) prior to antibody-mediated SVV pull-down, or no nanobody was added (lane 4, positive control). Hsp90AA1 only co-precipitates when beads are specifically enriched for SVV (lane 4). SVVNb8 does not affect the interaction between Hsp90AA1 and SVV (lane 2), nor does GFPNb (lane 1). Only SVVNb8 co-precipitates with the complex (lane 2). Lane 5 and lane 6 contain 1 μg of GFPNb and SVVNb8, respectively. An anti-His_6_ antibody was used to visualize the nanobodies. Longer exposure time was needed to detect SVV and Hsp90AA1 in crude lysate (CL) than in other conditions. Full-length blots are presented in Supplementary Figure S4. (**d**) XTT cell viability assay on HeLa cells transiently expressing EGFP (control) or SVVNb8-EGFP. Graphs represent mean net absorbance of formazan dye (dotted line) with range (three times three independent measurements; n = 3). P_0.05_-value was determined by a Mann-Whitney Rank Sum Test. SVVNb8-EGFP expression does not significantly alter cell viability compared to EGFP expression (p = 0.40).

SVV plays a key role during different phases of mitosis as a member of the chromosomal passenger complex (CPC)^10^. This is reflected by the presence of the protein in the spindle midzone and midbody in HeLa cells during late anaphase and telophase respectively (Fig. 3a). Upon SVVNb8 expression, the nanobody signal colocalizes with SVV at the spindle midzone and midbody (Fig. 3b, arrowheads). SVVNb8 is also enriched between sister chromatids and at cell poles (Fig. 3b, arrows), while this is not the case for the commercial antibody (Fig. 3a). SVV RNAi completely abolishes this SVVNb8 pattern (see Supplementary Figure S1). Subsequently, we investigated if SVVNb8 impedes the interaction of SVV with the chaperone Hsp90AA1, which is necessary for SVV stability^34^. SVVNb8 did not interfere with the SVV/Hsp90AA1 interaction and co-precipitated with the complex in MDA-MB-231 cells (Fig. 3c). SVV stability in SVVNb8 presence is supported by the fact that we did not observe aberrant cell division in SVVNb8-expressing cells (Fig. 3b). Moreover, XTT cell viability assays show that SVVNb8-EGFP expression does not have a significant effect on cell viability when compared to an EGFP-control (p = 0.40) (Fig. 3d). In brief, we conclude that SVVNb8 accurately traces SVV at the CPC during late anaphase and telophase and does thereby not affect cell division or cell viability.

### SVVNb8 efficiently delocalizes endogenous SVV towards various cell organelles

#### Nuclear export and import

**Figure 4:**
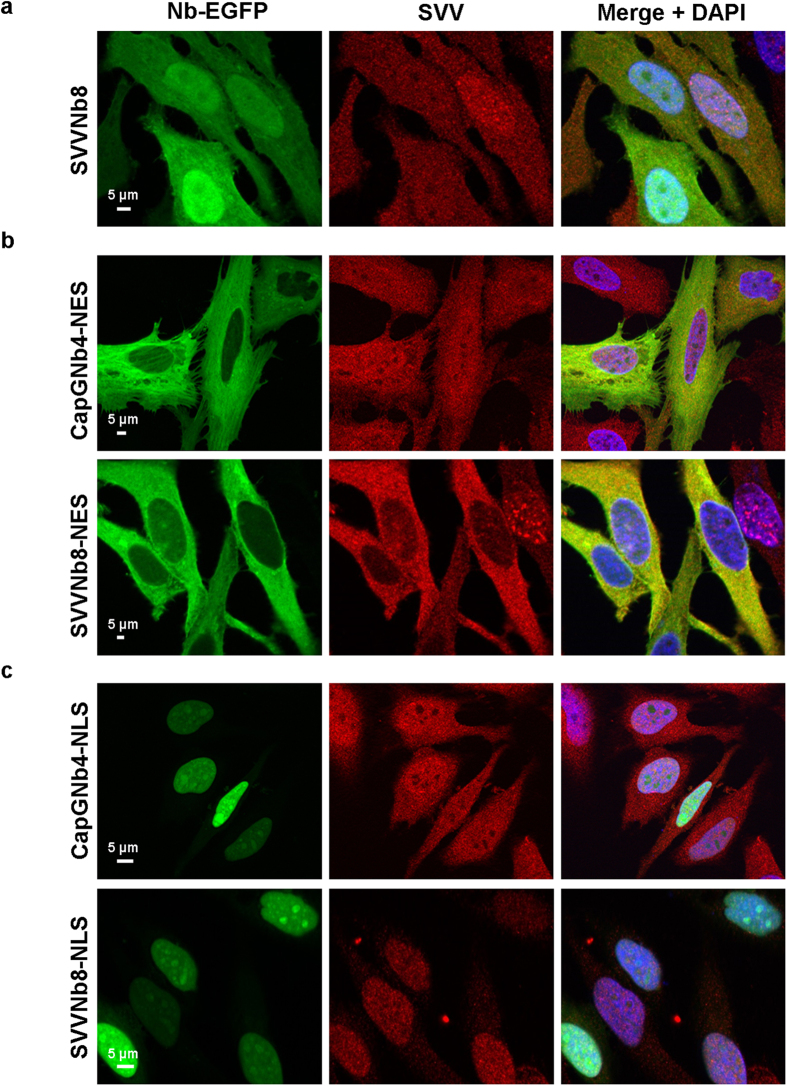
SVVNb8 redirects endogenous SVV in and out of the nucleus. Representative epifluorescence images of HeLa cervix cancer cells transiently expressing EGFP-tagged SVVNb8 without (**a**) or with an nuclear export signal (**b**) or nuclear localization signal (**c**). CapGNb4 was used as a negative control. The nanobody and endogenous SVV are distributed over the whole cell body (**a**). The nuclear export signal (NES) excludes both nanobodies from the nucleus, with only SVVNb8-NES resulting in delocalization of SVV (**b**). The nuclear localization signal (NLS) guides both nanobodies towards the nucleus, but only SVVNb8-NLS results in nuclear import of SVV (**c**). Nuclei are visualized with DAPI.

To further ascertain the efficacy of SVVNb8 as intrabody and explore its ability to bind and relocalize its target to different cellular compartments, we coupled EGFP-SVVNb8 C-terminally with a nuclear export signal (NES) or nuclear localization signal (NLS). The signal sequences are derived from the NES of MAPKK^35^ and the NLS from the Simian virus 40 large T antigen^36^, respectively (see Supplementary Table S1). SVVNb8 and SVV are homogenously distributed throughout HeLa cancer cells during interphase and to some extent enriched at the nucleus (Fig. 4a). Tagging with NES or NLS delocalizes both the control CapG nanobody and SVVNb8 to the cytoplasm (Fig. 4b, left panels) or nucleus (Fig. 4c, left panels), respectively. Only SVVNb8 is however able to achieve SVV nuclear export or import (Fig. 4b,c, middle panels). We already reported successful CapG delocalization towards the nucleus or cytoplasm by CapGNb4-NLS or CapGNb4-NES, respectively^25^. Thus, NES and NLS-tagged SVV intrabodies are able to efficiently guide the SVV pool out, or into the nucleus, respectively.

#### Mitochondrial membrane anchoring

**Figure 5:**
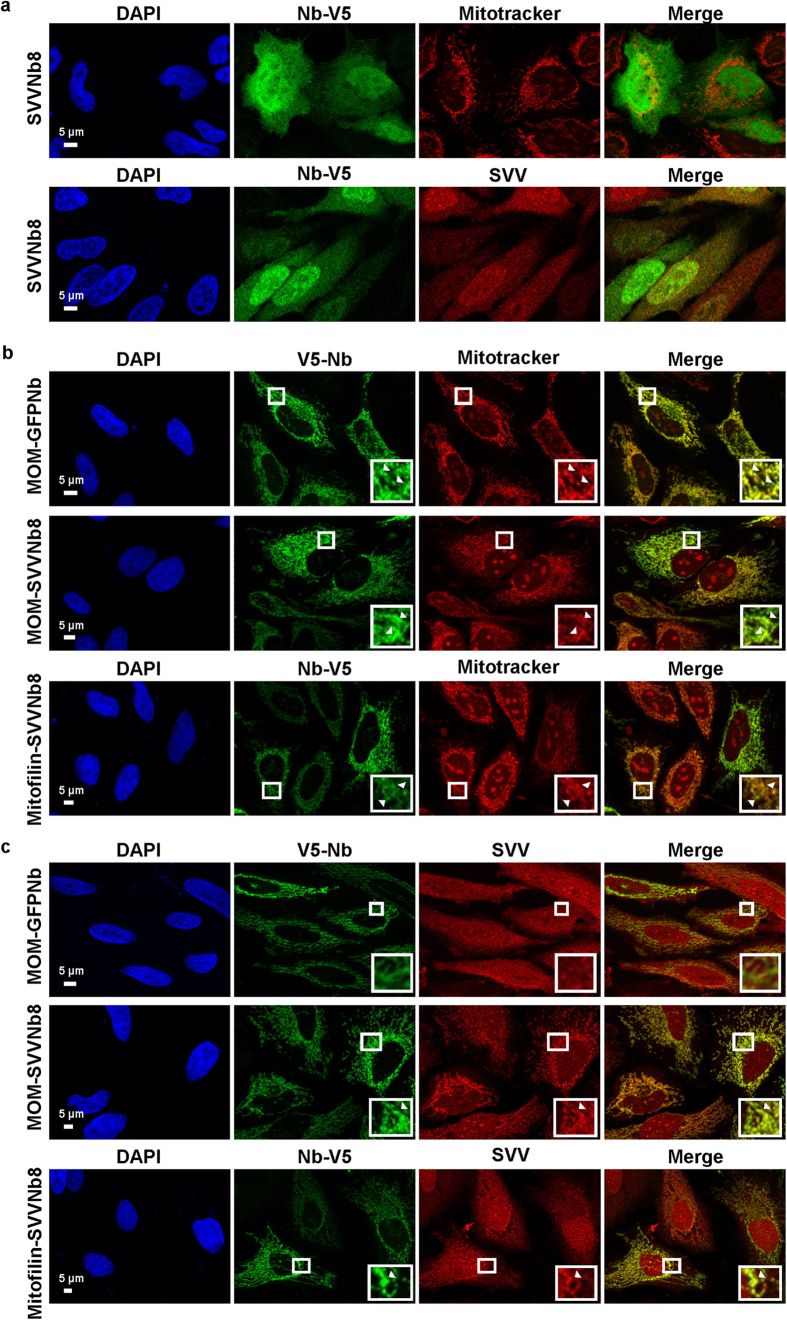
SVVNb8 captures endogenous SVV at mitochondria. Representative epifluorescence images of HeLa cervix cancer cells transiently expressing SVVNb8 (**a**) equipped with a MOM or mitofilin-tag (**b**,**c**). MOM-tagged GFP nanobody was used as a negative control. The SVV nanobody and SVV are distributed throughout the cell without enrichment at the mitochondria (**a**). MOM or mitofilin-tagged control or SVV nanobodies become enriched at the mitochondria, labelled with Mitotracker (**b**). However, SVV only adopts a mitochondria-like pattern when MOM or mitofilin-tagged SVVNb8 is expressed (**c**). Nuclei were visualized with DAPI and nanobodies with anti-V5 antibody. Boxed areas are enlarged in the bottom right insets and arrowheads Δ indicate areas of colocalization.

We next set out to immobilize endogenous SVV at the outer mitochondrial membrane or in the inner mitochondrial space. To this end, an N-terminal mitochondrial outer membrane (MOM) or mitofilin-tag was coupled to V5-tagged SVVNb8. The MOM-tag originates from yeast TOM70^37^ while the mitofilin-tag is derived from human mitofilin^38^ (Supplementary Table S1). SVVNb8 is normally not enriched at Mitotracker-labelled mitochondria of HeLa cells, but is ubiquitously distributed together with SVV with slight enrichment at the nucleus (Fig. 5a). Conversely, tagging nanobodies with a MOM or mitofilin sequence efficiently redirects both control GFP nanobody and SVVNb8 towards mitochondria (Fig. 5b). Moreover, SVV does not adopt a mitochondrial-like pattern in MOM-GFPNb expressing control cells, while MOM or mitofilin-tagged SVVNb8 clearly relocalizes SVV (Fig. 5c). Confocal microscopy allowed a more detailed investigation of this SVV-MOM/mitofilin-SVVNb8 mitochondrial recruitment (See Supplementary Figure S2). MOM-SVVNb8 and relocalized SVV typically encircle the mitochondrial pattern and the same applies to mitofilin-SVVNb8 with SVV. Examination of the intensity profiles over the mitochondria confirms this characteristic outer rim enrichment of the nanobody and SVV, as their intensity peaks at the edges of the Mitotracker peak. In conclusion, MOM and mitofilin-tagged SVV intrabodies are able to efficiently capture endogenous SVV at mitochondria and both accumulate at the outward mitochondrial area. A schematic representation of the mitochondrial recruitment assay is depicted in Supplementary Figure S2.

#### Peroxisomal matrix transport

**Figure 6:**
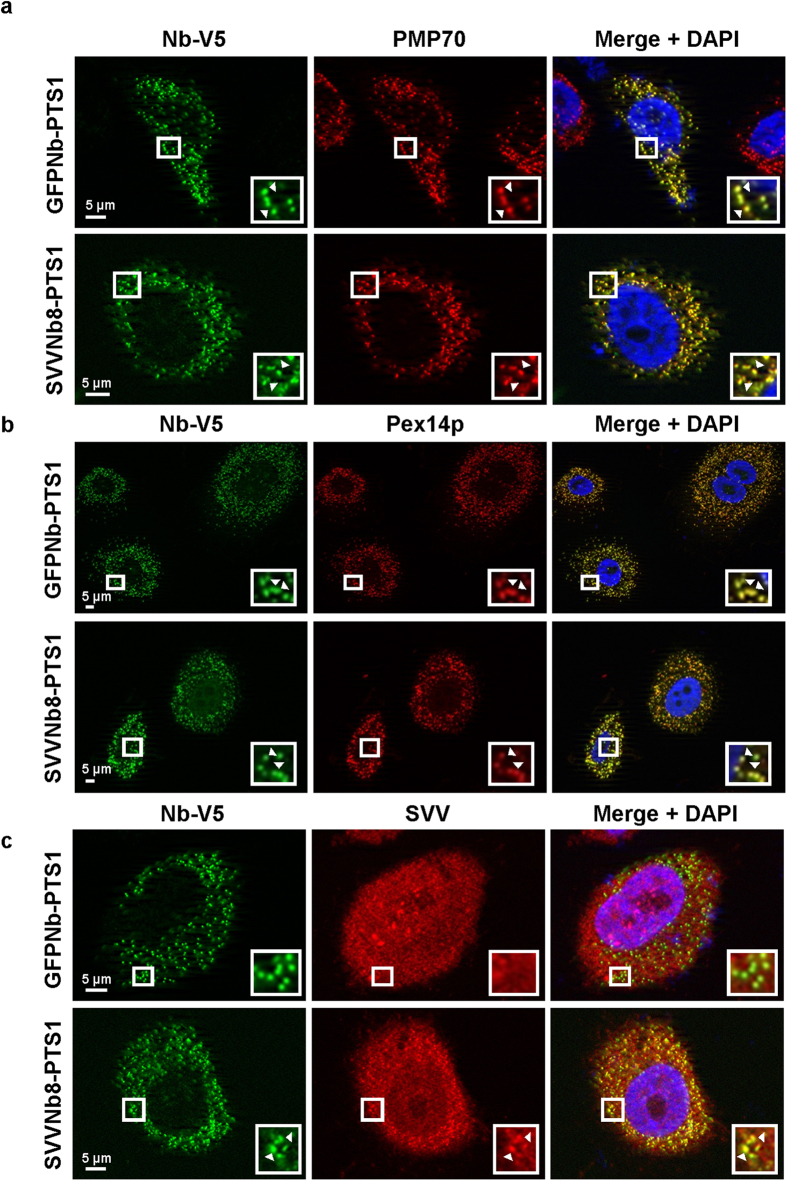
The PTS1-tagged nanobody redirects SVV towards the peroxisomes. Representative epifluorescence images of PC-3 prostate cancer cells transiently expressing PTS1-tagged GFP nanobody (control) or SVVNb8. Both nanobodies colocalize with the peroxisomal markers PMP70 (**a**) and Pex14p (**b**). Only SVVNb8-V5/PTS1, but not the control nanobody, is able to induce SVV delocalization to a peroxisome-like pattern (**c**). Nuclei are visualized with DAPI and nanobodies with anti-V5 antibody. Boxed areas are enlarged in the bottom right insets and arrowheads Δ indicate areas of colocalization.

Finally, SVVNb8 was coupled to a consensus sequence of the type I peroxisomal targeting sequence (PTS1)^39^, with the purpose of shuttling the nanobody to the peroxisomal matrix (Supplementary Table S1). The fluorescence signal of PTS1-tagged control GFPNb-V5 and SVVNb8-V5 adopts a dotted pattern in PC-3 prostate cancer cells, which colocalizes with the peroxisome markers PMP70 (Fig. 6a) and Pex14p (Fig. 6b). This indicates that both nanobodies are transported towards the peroxisomes. Colocalization between the nanobody signal and SVV in a dotted pattern only occurs upon PTS1-tagged SVVNb8 expression (Fig. 6c). In contrast, SVV is virtually ubiquitous in the control PTS1-tagged GFPNb cells, where it resides in the nucleus as well as in the cytoplasm.

Of note, peroxisomal transport of the nanobodies is Pex5p-dependent, since Pex5p-deficient fibroblasts only succeed in nanobody transport when the Pex5p import receptor is co-expressed (see Supplementary Figure S3). Consequently, Pex5p expression induces a speckled SVV pattern in Pex5p-deficient fibroblasts, which colocalizes with SVVNb8 and Pex5p. This suggests that the complete SVV-SVVNb8 complex is transported to the peroxisomes by means of the PTS1-tag and that this process is Pex5p-dependent.

**Figure 7:**
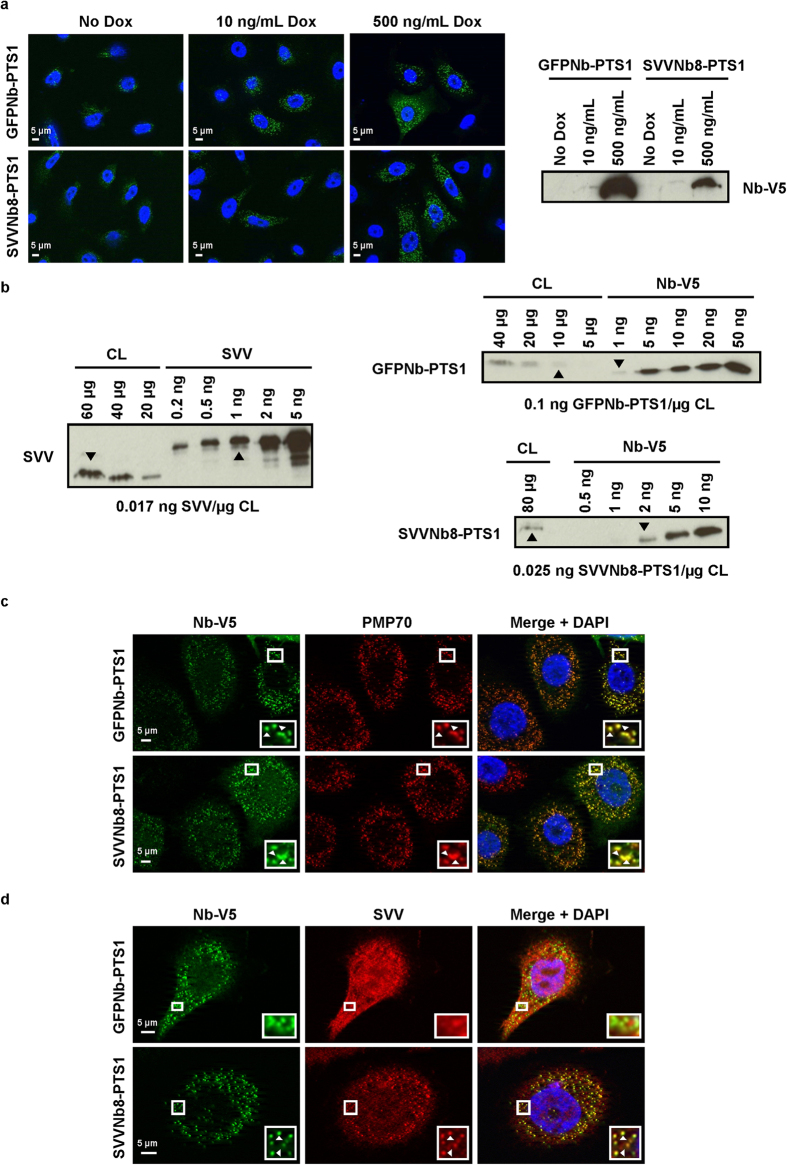
Characterization of stable PC-3 cell lines with Dox-inducible expression of PTS1-tagged GFP nanobody or SVVNb8. (**a**) Representative epifluorescence images of stable PC-3 cells upon addition of 0 ng/mL, 10 ng/mL or 500 ng/mL Dox for 24 h showing inducibility and concentration-dependence of PTS1-tagged nanobody expression (left). Nuclei are visualized with DAPI and nanobodies with anti-V5 antibody. At the right, a Western blot is depicted, confirming inducibility and concentration-dependence of PTS1-tagged nanobody expression. Full-length blot is presented in Supplementary Figure S4. (**b**) Determination of the amount of nanobody and endogenous SVV in the generated stable cell lines induced with 500 ng/mL Dox by comparing Western blot analysis on crude lysate (CL) with recombinant standards (His_6_-SVV and LPLNb5-V5, a nanobody targeting the actin-bundling protein L-plastin). Nanobodies were blotted with anti-V5 antibody. Arrowheads Δ indicate the estimated amount of protein corresponding to the level in the lysate, resulting in the ratios depicted at the bottom. Approximately six times more PTS1-tagged GFP nanobody is present compared to endogenous SVV. Conversely, PTS1-tagged SVVNb8 is expressed at an approximately 1:1 molar ratio to endogenous SVV. Full-length blots are presented in Supplementary Figure S4. (**c**) Representative epifluorescence images of PC-3 prostate cancer cells stably expressing PTS1-tagged GFP nanobody (control) or SVVNb8 upon induction with 500 ng/mL Dox. Both the control nanobody and SVVNb8-V5/PTS1 colocalize with the peroxisomal marker PMP70. (**d**) Only SVVNb8-V5/PTS1 is able to delocalize SVV towards the peroxisomes. Nuclei are visualized with DAPI and nanobodies with anti-V5 antibody. Boxed areas are enlarged in the bottom right insets and arrowheads Δ indicate regions of colocalization.

Next, stable PC-3 cell lines with doxycycline-inducible GFPNb-V5/PTS1 or SVVNb8-V5/PTS1 expression were generated. This allows fine-tuning of nanobody expression and consistent expression levels, which increases reproducibility. No expression leakage was observed in the stable cell lines and maximal expression is achieved by incubation with 500 ng/ml doxycycline (Dox) (Fig. 7a). We evaluated the amount of SVV and PTS1-tagged nanobody expressed in the latter condition by comparing different amounts of crude lysate with recombinant standards (Fig. 7b). GFPNb-PTS1 is approximately six times more expressed than endogenous SVV and there are roughly equal amounts of SVVNb8-PTS1 and SVV. Similarly as in transiently transfected cells, the PTS1-tagged nanobodies colocalize with the peroxisome marker PMP70 (Fig. 7c) and only SVVNb8-PTS1 relocates endogenous SVV (Fig. 7d).

**Figure 8:**
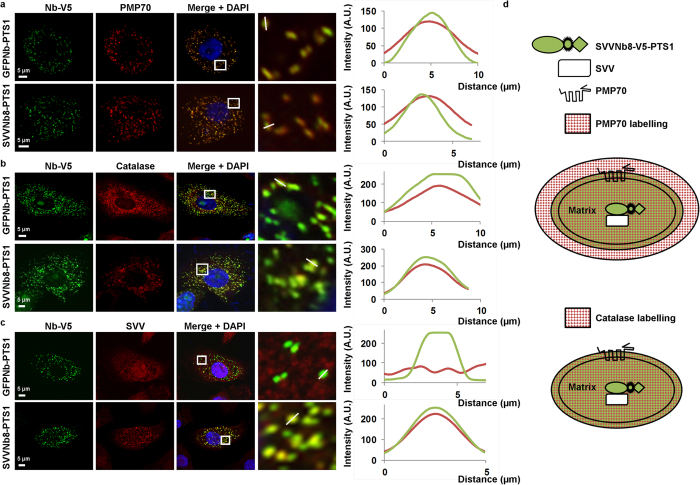
SVVNb8 localizes at the peroxisomal matrix and redistributes SVV accordingly. Representative confocal images of PC-3 prostate cancer cells expressing PTS1-tagged GFP nanobody (control) or SVVNb8 upon 24 h induction with 500 ng/mL Dox. Both the control and SVVNb8 colocalize with the peroxisomal marker PMP70 (**a**), although PMP70 is additionally enriched at a region surrounding the nanobody. Colocalization studies with catalase on the other hand, reveal intensity overlap with the nanobody (**b**). Colocalization of SVV with the GFP nanobody does not occur, but is complete with the SVVNb8 (**c**). Nuclei are visualized with DAPI and nanobodies with anti-V5 antibody. Boxed areas are enlarged at the right. Graphs represent corresponding intensity profiles generated through the white solid lines. (**d**) Schematic representation of the different components at the peroxisomes. PMP70 is a transmembrane protein and its signal colocalizes with and additionally surrounds the nanobody signal. Catalase on the other hand is an internal peroxisomal component and colocalizes completely with SVVNb8. SVVNb8 signal colocalizes with the SVV signal.

Peroxisomal transport was investigated in more detail by confocal microscopy and intensity profiles along peroxisomes in these stable cell lines. Concerning the integral peroxisomal membrane protein PMP70, the margins of the intensity profile reveal a more expanding PMP70 signal compared to the nanobody signal and both peak simultaneously (Fig. 8a). This suggests that the PTS1-tagged nanobodies are localized under, or at least at, the peroxisomal membrane. Secondly, the signal of the genuine peroxisomal matrix protein catalase starts, peaks and ends concurrently with the nanobody signal along the peroxisome (Fig. 8b). Thus, the nanobody will likely be in, or at least in close proximity of, the peroxisomal matrix. Finally, the signal intensity course for SVV and PTS1-tagged SVVNb8 is identical (Fig. 8c), suggesting SVV relocalization towards places of nanobody enrichment. A schematic representation of the peroxisomal transport assay is depicted in Fig. 8d.

In summary, coupling SVVNb8 to the peroxisomal matrix targeting sequence PTS1 was sufficient to successfully delocalize the nanobody and SVV towards peroxisomes in a Pex5p-dependent manner. Moreover, confocal microscopy suggests peroxisomal import of the PTS1-tagged SVVNb8 and a redistribution of SVV towards this location.

## Discussion

In this study, we have generated a SVV nanobody (SVVNb8) that binds SVV with high affinity. Expressed as intrabody, SVVNb8 correctly tracks SVV during different phases of mitosis, without interfering with SVV function. Furthermore, coupling SVVNb8 with a delocalization tag efficiently delocalizes endogenous SVV towards the nucleus and peroxisomes, and captures SVV at mitochondria. Based on our findings, we believe that SVVNb8 is an excellent tool to further elucidate SVV biology and topography.

Although Nb2, 3, 4, 6, 7 and 16 were successfully expressed recombinantly, they were not able to bind endogenous SVV (Fig. 1a,b). These nanobodies likely recognize an epitope encompassing His_6_ and SVV, or bind SVV with very low affinity. Nb1, 5, 18, 23 and 26 bind their target when recombinantly expressed, but not as an intrabody (Fig. 1). This is quite exceptional in our experience. Possibly, these nanobodies suffer from the reducing intracellular environment, which may theoretically lead to reduction of disulphide bridges, partial unfolding and thereby loss-of-function.

SVVNb8 most likely detects a conformational epitope on SVV, as the nanobody cannot detect denatured SVV on blot (Fig. 2b). Similarly, L-plastin nanobodies bind conformational epitopes and also failed to detect denatured L-plastin^32^. By contrast, a nanobody against gelsolin (gelsolin Nb13) recognizes a linear linker between two gelsolin subdomains and can be used as a detection tool in Western blot^40^.

SVV function is of key importance during different stages of mitosis^4^,^5^,^6^,^7^. The subcellular localization of SVV during mitosis has been contentious, as researchers only detected a pool of SVV associated with tubulin (microtubule and centrosomes)^4^, or a pool of SVV associated with the chromosomal passenger complex (CPC)^9^ *in vivo*. We found that intrabody SVVNb8 was enriched at the central spindle midzone and midbody during late anaphase and telophase (Fig. 3b, arrowheads), which is reminiscent of the CPC pattern. Moreover, SVVNb8 was enriched at cell poles and between sister-chromatids in a threadlike pattern, presumably detecting microtubule and centrosome-associated SVV (Fig. 3b, arrows). The microtubule-associated SVV staining is however rather blurry, which could be explained by the fact that (I) we did not stabilize microtubules during immunostaining^4^,^41^ and (II) SVVNb8 is highly expressed. The latter can be solved by using inducible stable cell lines where nanobody expression can be fine-tuned (cf. Fig. 7). SVV RNAi confirms SVVNb8 specificity as the typical SVVNb8 pattern disappears when SVV siRNA is expressed (Supplementary Figure S1). Since SVVNb8 intrabody labels both SVV subpopulations, we conclude that the nanobody acts as a faithful SVV tracer. This is in line with one of our previous reports on nuclear transport factor 2 (NTF2) nanobodies, where we were able to reveal a new location of NTF2 at the centrosome^42^. Recently, fluorescently labelled anti-tubulin nanobodies were used to visualize microtubules in super-resolution microscopy. Nanobodies are 10× smaller compared to conventional antibodies and accordingly, the distance between fluorophore and target is significantly reduced. In this way, individual microtubules could be visualized^43^. As SVVNb8 reliably binds its target, we believe that SVVNb8 is a potential candidate to visualize endogenous SVV in super-resolution microscopy.

Interference with SVV expression or function causes an increase in apoptosis^5^,^7^,^44^, particularly in cells approaching mitosis^44^. The apoptotic response results from mitotic catastrophe, caused by multiple mitotic defects after SVV knock down^7^. Here, we show that SVVNb8 expression does not affect cell division or cell viability (Fig. 3b,d), which implicates that the nanobody does not interfere with SVV function during mitosis. Moreover, we presume that SVV protein stability is preserved in the presence of SVVNb8, as the nanobody does not inhibit SVV to interact with its chaperone Hsp90AA1 (Fig. 3c). We and others have already used nanobodies in live cell imaging, to study endogenous antigen localization *in vivo*^28^,^45^,^46^,^47^. Based on these observations, we believe that SVVNb8 can be a good tracer for endogenous SVV in live cell imaging to further investigate the complex topography of the protein.

The epitope of SVVNb8 could not be pinpointed, but our results suggest that the BIR-domain (without the Hsp90AA1-interacting residues K79-K90^34^) is involved. The α-helix and dimer interfaces are involved in extensive protein-protein interactions required for proper SVV function during mitosis (tubulin binds the α-helix^2^,^4^, CPC members Borealin and INCENP bind the α-helix and dimer interfaces^10^,^48^), which is not hampered when expressing the nanobody intracellularly. Furthermore, SVVNb8 does not interfere with SVV dimerization (Fig. 2c), which requires the dimer interfaces^2^. As SVVNb8 did not recognize BIR or BIR-dimer interfaces in a GST-pull down epitope mapping (Fig. 2a), the conformation of the epitope is probably altered in truncated SVV.

We equipped intrabody SVVNb8 with different delocalization tags in order to demonstrate nanobody functionality *in vivo* and to manipulate endogenous SVV localization. The preferential site for nanobody fusion is C-terminal, as this is the natural connection site for the constant domain. In addition, nanobody paratope clusters at the N-terminal end^30^; adding a delocalization tag therefore potentially interferes with antigen binding. Here we show that SVVNb8 retains its antigen binding activity upon fusion of N-terminal tags (mitofilin or MOM), as SVV faithfully follows the location of tagged SVVNb8 (Fig. 5c and Supplementary Figure S2).

Tagging SVVNb8 with a MOM or mitofilin tag is sufficient to delocalize the nanobody towards the mitochondria (Fig. 5b and Supplementary Figure S2) and induces mitochondrial enrichment of SVV (Supplementary Figure S2). Intensity profiles confirm MOM/mitofilin-SVVNb8 and SVV accumulation at the outer rim of the mitochondria (Supplementary Figure S2), but limited Mitotracker and/or confocal microscope resolution does not allow distinction between outer membrane (MOM-delocalization) or inner membrane (mitofilin-delocalization) anchored SVV-SVVNb8-complex. In other words, unlike the peroxisomal transport assay, we are unable to exactly pinpoint the location of the protein complex based on the intensity profiles (Supplementary Figure S2). Mitotracker is expected to accumulate at the mitochondrial matrix, where it covalently binds free thiol groups^49^. We assume that the dye partially reacts with proteins in the intermembrane space and/or at the outer membrane, resulting in a less well-defined Mitotracker signal. Moreover, it is not possible to distinguish inner from the outer mitochondrial membrane when using a confocal microscope. Nevertheless, John and co-workers reported successful mitochondrial import of proteins coupled to amino acids 1–187 (containing the mitochondrial targeting and membrane anchor domain) of mouse mitofilin^38^. We believe that this mitochondrial recruitment assay, using mitofilin-tagged nanobodies, can be used as a model to study mitochondrial protein import.

Unlike mitochondria and endoplasmic reticulum, peroxisomes are capable of importing oligomeric protein complexes^50^,^51^,^52^,^53^,^54^,^55^, although the physiological relevance of oligomeric import has been questioned^55^. Nevertheless, our results strongly suggest peroxisomal import of a protein complex and therefore support previous findings. Since SVVNb8-PTS1 is likely imported into the peroxisomes (Fig. 8a,b, intensity profiles) and the SVVNb8-PTS1 signal colocalizes with the SVV signal (Fig. 8c, intensity profile), we expect that SVV is also imported into peroxisomes. We also confirm that tagging one of two interacting proteins with PTS1 is sufficient to mediate import of the other^50^,^51^,^52^,^53^,^54^,^55^.

Altogether, these findings show that we can manipulate endogenous SVV location using tagged SVVNb8. Delocalization can be an elegant strategy to perturb protein function by limiting free diffusion in cells and restricting protein availability at places where it is needed^42^,^56^. For instance, tumour cells harbour a mitochondrial pool of SVV which, in contrast to cytosolic SVV, protects cells from caspase-dependent and independent apoptosis^3^,^11^,^12^,^13^,^14^,^15^,^57^. Mitofilin-SVVNb8 could be used to further study the anti-apoptotic function of mitochondrial SVV. When mitochondrial SVV is immobilized at the inner membrane, we predict that it can no longer participate in the cytoprotective response and tumour cells are expected to become more sensitive to apoptotic stimuli.

Hypothetically, protein delocalization can also be technically applied in organellar proteomics, in order to tackle high background when identifying protein interaction partners. Organellar proteomics implicates subcellular fractionation of the organelle of interest before mass spectrometry, which eliminates potentially contaminating cytoplasmic proteins. Sample complexity is therefore compatible with the sensitivity of current mass spectrometers and allows identification of low abundance proteins^58^. Supposing SVV is imported into peroxisomes by means of SVVNb8-PTS1, we could potentially detect SVV interaction partners by organellar proteomics. Furthermore, protein catalogues of peroxisomes are available^59^,^60^, which makes it possible to discern bona fide peroxisomal proteins from SVV binders. Of note, we do not know the size limitations of the peroxisomal import machinery. It is possible that this technique is not suitable for large protein complexes, as import would be ineffective or impossible. Nonetheless, it is worth mentioning that tetrameric catalase, with a molecular weight of 240 kDa, was successfully imported into the peroxisomal matrix^50^.

In summary, we show that SVVNb8 is a versatile and excellent tool to further sort out the complex SVV biology and topography. Moreover, we believe that this nanobody technology presented here can be easily extrapolated to other (structural) proteins.

## Methods

### Generation of survivin (SVV) nanobodies

See Supplementary Methods for details on the generation of survivin-specific nanobodies.

### cDNA cloning

See Supplementary Methods for a detailed description on the cloning reactions.

### Antibodies

See Supplementary Methods for a detailed description on the antibodies used in this study.

### Production and purification of recombinant SVV nanobodies, GST-SVV, GST-SVV fragments, GST-cortactin and His_6_-SVV

See Supplementary Methods for details on recombinant protein expression and purification.

### SVV RNAi

See Supplementary Methods for a detailed description on the SVV RNAi experiment.

### Cell culture, transfection and transduction

HeLa, HEK293T, MDA-MB-231 and PC-3 cells were grown at 37 °C in a humidified 10% CO_2_ incubator. Pex5p-deficient fibroblasts (Zellweger cells) were a kind gift of Prof. Dr. Ronald Wanders (Department of Clinical Chemistry, Academic Medical Centre, University of Amsterdam, The Netherlands) and were maintained at 37 °C and 5% CO_2_. All cells were cultured in DMEM, PC-3 cells were cultured in RPMI (media from Gibco, Thermo Fisher Scientific, Waltham, MA, USA) supplemented with 10% foetal bovine serum. Transient transfection was generally achieved with jetPRIME (Polyplus Transfection Inc., New York, NY, USA). HEK293T cells were transfected using the calcium phosphate method. Transfection in Pex5p-deficient fibroblasts was performed using the Neon Transfection System (Thermo Fisher Scientific). Cells were either sequentially transfected (Pex5p-FLAG transfection 48 h after nanobody transfection) or cotransfected.

PC-3 cells stably expressing GFPNb-V5-PTS1 or SVVNb8-V5-PTS1 were created using the Lenti-X Tet-On Advanced Inducible Expression System (Clontech) as previously described^56^. In brief, HEK293T were calcium phosphate-transfected with packaging (psPAX2), envelope (pMD2.G) and nanobody (pLVX-Tight-Puro) plasmids. Medium with lentiviral particles was collected 48 h and 72 h post-transfection, filtered (0.45 μm) and concentrated by means of ultracentrifugation. Viral titre was determined by crystal violet staining on transduced and puromycin (nanobody) or G418 (regulator) -selected HEK293T cells. Next, PC-3 cells were infected at an MOI = 5 with regulator and nanobody virus and subjected to puromycin and G418 selection for 2 weeks.

### Assessing SVV nanobody binding characteristics

#### Pull-down and co-immunoprecipitation assays

HeLa, MDA-MB-231 or transfected HEK293T cells were lysed with ice-cold lysis buffer (0.5% NP-40, 1 mM PMSF and 1 mM protease inhibitor cocktail in PBS), sonicated and pelleted. Protein concentrations were determined using the Bradford assay (Bio-Rad Laboratories, Hercules, CA, USA). For the pull-down experiment, recombinant HA-tagged nanobodies from periplasmic extract were immobilized onto HA-agarose beads (Sigma-Aldrich, St. Louis, MO, USA) (1–2 h at 4 °C), followed by incubation with 1 mg crude HeLa lysate (1 h at 4 °C). For the SVV-nanobody co-immunoprecipitation experiment, EGFP-tagged intrabodies from 1 mg HEK293T lysate were immobilized onto anti-GFP antibody (1–2 h at 4 °C), followed by coupling to protein G sepharose beads (GE Healthcare) (1 h at 4 °C). For the SVV-Hsp90 co-immunoprecipitation experiment, 1 mg crude MDA-MB-231 lysate was pre-incubated with 5 μg purified GFPNb-His_6_/STREP or SVVNb8-His_6_/STREP for 1 h at 4 °C, followed by incubation with anti-SVV antibody (D-8) and protein G sepharose beads (GE Healthcare) (1 h each at 4 °C). After washing, beads were eluted by boiling in Laemmli SDS sample buffer (65 mM Tris-HCl pH 6.8, 20% glycerol, 5% SDS, 0.2% bromophenol blue, 5% β-mercaptoethanol in Milli-Q), followed by SDS-PAGE and Western blot analysis. Ab469 was used to detect SVV on blot.

#### Epitope mapping

Glutathione beads coated with GST-fusion proteins were incubated with 10 μg purified recombinant SVVNb8-V5/SBP/His_6_ in wash buffer (0.5% Triton X-100, 1 mM PMSF and 1 mM protease inhibitor cocktail in PBS) during 2 h at 4 °C. Beads were recovered by centrifugation and the samples were subsequently washed, boiled, fractionated by SDS-PAGE and visualized by Coomassie Blue staining.

#### Nanobody-mediated detection of endogenous SVV on Western blot

Equal amounts of HEK293T, HeLa and PC-3 lysates were analysed using SDS-PAGE and blotted onto a nitrocellulose membrane. After 1 h in blocking solution (5% non-fat dry milk in 100 mM Tris pH 7.5, 1.5 M NaCl and 1% Tween (TBS-T)), the membranes were incubated with 5 μg/ml purified SVVNb8-His_6_/STREP, 5 μg/ml purified GFP Nb-His_6_/STREP or 1 μg/ml commercial SVV antibody (ab469) (overnight, at 4 °C). Bound His_6_-tagged nanobody was detected by subsequent incubation with anti-His_6_ antibody and anti-mouse HRP-coupled antibody (1 h incubation each at room temperature). Detection of the commercial anti-SVV antibody was performed using anti-rabbit HRP-coupled antibody (1 h at room temperature).

#### Isothermal titration calorimetry

The binding affinity of SVVNb8-His_6_/STREP to GST-SVV was determined by isothermal titration calorimetry, using a Microcal VP-ITC MicroCalorimeter (Malvern Instruments Ltd, Malvern, UK) as previously described^40^. 45 μM purified SVVNb8-His_6_/STREP was gradually titrated within the sample cell containing 4.5 μM GST-SVV, after dialysis against 20 mM Tris pH 7.5, 150 mM NaCl. Heat release was registered per SVVNb8-His_6_/STREP injection in function of time. Each peak was integrated and plotted versus the molar ratio of SVVNb8-His_6_/STREP to GST-SVV. The ITC data was fitted to a ‘One Set of Sites’ model using the Microcal Origin software, from which the affinity and stoichiometry of the reaction could be derived.

#### XTT assay

Equal amounts of HeLa cells were seeded in a 96-well plate and transiently transfected with EGFP only or EGFP-tagged SVVNb8. 24 h post-transfection, an XTT assay (XTT cell proliferation kit II, Roche) was performed according to the manufacturer’s instructions. Briefly, 50 μl of XTT labelling mixture was added to each well. At time points 0 h and 4 h, absorbance was measured at 450 nm (A_formazan_) and 620 nm (A_background_). Background absorbance was subtracted from formazan absorbance at each time point. Net absorbance (A_4 h_ – A_0 h_) is proportionate to the amount of metabolically active cells. The effect of each construct was studied three times in three independent experiments. A Mann-Whitney Rank Sum Test (ordinal and independent observations which are not normally distributed; n = 3, pEGFP-N1 versus SVVNb8-EGFP transfected cells, α = 0.05, two-tailed) was used to analyse the results via Sigmaplot (Systat Software Inc., San Jose, CA, USA).

### Native PAGE

0.5 μg recombinant His_6_-SVV, GST-SVV and GST-cortactin in native sample buffer (Laemmli SDS sample buffer without SDS and β-mercaptoethanol) was loaded on top of a 15% or 6% native polyacrylamide gel, which lacks SDS. Protein mono- and oligomers were analyzed by Western blot.

### Immunofluorescence and microscopy

Coverslips were coated with 50 μg/ml rat tail type I collagen (BD Biosciences, Franklin Lakes, NJ, USA) in PBS (with Ca^2+^ and Mg^2+^) for 1 h at 37 °C. Cells were seeded onto the coated coverslips at a density of 60–80%. 24 h post-seeding, cells were transfected with nanobody-plasmid or nanobody expression was induced by adding doxycycline to a final concentration of 500 ng/ml, unless mentioned otherwise. Immunostaining was performed 24 h later. Pex5p-deficient fibroblasts were seeded onto collagen-coated coverslips directly after Neon transfection. Visualization of mitochondria was achieved by incubating living cells with 0.1 μM Mitotracker Orange (M-7510, Thermo Fisher Scientific) in cell-specific serum-free medium for 30 min at 37 °C. Cells were fixed with 3% paraformaldehyde for 25 min, permeabilized 5 min with 0.2% Triton X-100 and additionally incubated with 0.75% glycine for 20 min. Every incubation step was followed by at least three washing steps. After blocking in 1% bovine serum albumin (BSA), coverslips were incubated with primary and secondary antibodies (resp. 1 h at 37 °C and 30 min at room temperature, both diluted in 1% BSA). Antibody incubation steps were separated by four 4 min wash steps in 1% BSA. Nuclei were stained with 0.4 μg/ml DAPI (D9542, Sigma-Aldrich) during the last antibody incubation step. Finally, cells were washed twice. PBS with Ca^2+^ and Mg^2+^ was used for all dilutions and washing steps, unless mentioned otherwise. All SVV-immunostainings were performed using ab469. Coverslips were mounted onto microscope slides using Vectashield antifade mounting medium (Vector Laboratories, Burlingame, CA, USA) and were sealed with nail polish. Cells were analyzed at room temperature using a Zeiss Axiovert 200 M fluorescence microscope with Apotome module (Zeiss x63 1.4-NA Oil Plan-Apochromat objective, Carl Zeiss, Oberkochen, Germany) and Axiovision 4.5 software (Zeiss) or an Olympus IX81 Fluoview 1000 confocal laser scanning microscope (Olympus x60 1.36-NA Oil UplanSApo objective, Olympus, Tokyo, Japan) with FluoView FV 1000 software (Olympus). Intensity profiles were generated by means of ImageJ (National Institutes of Health, Bethesda, MD, USA).

## Additional Information

**How to cite this article**: Beghein, E. *et al*. A new survivin tracer tracks, delocalizes and captures endogenous survivin at different subcellular locations and in distinct organelles. *Sci. Rep.* **6**, 31177; doi: 10.1038/srep31177 (2016).

## Supplementary Material

Supplementary Information

## References

[b1] Ambrosini G., Adida C. & Altieri D. C. A novel anti-apoptosis gene, survivin, expressed in cancer and lymphoma. Nat. Med. 3, 917–921, 10.1038/nm0897-917 (1997). 9256286

[b2] Verdecia M. A. . Structure of the human anti-apoptotic protein survivin reveals a dimeric arrangement. Nat. Struct. Biol. 7, 602–608 (2000). 10876248 10.1038/76838

[b3] Pavlyukov M. S. . Survivin Monomer Plays an Essential Role in Apoptosis Regulation. J. Biol. Chem. 286, 23296–23307, 10.1074/jbc.M111.237586 (2011). 21536684 PMC3123095

[b4] Li F. Z. . Control of apoptosis and mitotic spindle checkpoint by survivin. Nature 396, 580–584 (1998). 9859993 10.1038/25141

[b5] Carvalho A., Carmena M., Sambade C., Earnshaw W. C. & Wheatley S. P. Survivin is required for stable checkpoint activation in taxol-treated HeLa cells. J. Cell Sci. 116, 2987–2998, 10.1242/jcs.00612 (2003). 12783991

[b6] Lens S. M. A. . Survivin is required for a sustained spindle checkpoint arrest in response to lack of tension. Embo J. 22, 2934–2947, 10.1093/emboj/cdg307 (2003). 12805209 PMC162159

[b7] Lamers F. . Knockdown of survivin (BIRC5) causes apoptosis in neuroblastoma via mitotic catastrophe. Endocr.-Relat. Cancer 18, 657–668, 10.1530/erc-11-0207 (2011). 21859926

[b8] Rosa J., Canovas P., Islam A., Altieri D. C. & Doxsey S. J. Survivin modulates microtubule dynamics and nucleation throughout the cell cycle. Molecular biology of the cell 17, 1483–1493 (2006). 16407408 10.1091/mbc.E05-08-0723 PMC1382334

[b9] Uren A. G. . Survivin and the inner centromere protein INCENP show similar cell-cycle localization and gene knockout phenotype. Curr. Biol. 10, 1319–1328, 10.1016/s0960-9822(00)00769-7 (2000). 11084331

[b10] Jeyaprakash A. A. . Structure of a Survivin-Borealin-INCENP core complex reveals how chromosomal passengers travel together. Cell 131, 271–285, 10.1016/j.cell.2007.07.045 (2007). 17956729

[b11] Dohi T., Beltrami E., Wall N. R., Plescia J. & Altieri D. C. Mitochondrial survivin inhibits apoptosis and promotes tumorigenesis. J. Clin. Invest. 114, 1117–1127, 10.1172/jci200422222 (2004). 15489959 PMC522254

[b12] Dohi T. . An IAP-IAP complex inhibits apoptosis. J. Biol. Chem. 279 34087–34090, 10.1074/jbc.C400236200 (2004). 15218035

[b13] Song Z. Y. . A single amino acid change (Asp 53 ->Ala53) converts survivin from anti-apoptotic to pro-apoptotic. Molecular biology of the cell 15, 1287–1296, 10.1091/mbc.E03-07-0512 (2004). 14699067 PMC363130

[b14] Marusawa H. . HBXIP functions as a cofactor of survivin in apoptosis suppression. Embo J. 22, 2729–2740, 10.1093/emboj/cdg263 (2003). 12773388 PMC156760

[b15] Liu T., Brouha B. & Grossman D. Rapid induction of mitochondrial events and caspase-independent apoptosis in Survivin-targeted melanoma cells. Oncogene 23, 39–48, 10.1038/sj.onc.1206978 (2004). 14712209 PMC2292403

[b16] Fukuda S. & Pelus L. M. Survivin, a cancer target with an emerging role in normal adult tissues. Mol. Cancer Ther. 5, 1087–1098, 10.1158/1535-7163.mct-05-0375 (2006). 16731740

[b17] Xia F. & Altieri D. C. Mitosis-independent Survivin gene expression *in vivo* and regulation by p53. Cancer Res. 66, 3392–3395, 10.1158/0008-5472.can-05-4537 (2006). 16585159

[b18] Chu X. Y. . Overexpression of survivin is correlated with increased invasion and metastasis of colorectal cancer. J. Surg. Oncol. 105, 520–528, 10.1002/jso.22134 (2012). 22065492

[b19] Li Y. Y., Ma X. L., Wu X., Liu X. X. & Liu L. Prognostic Significance of Survivin in Breast Cancer: Meta-analysis. Breast J. 20, 514–524, 10.1111/tbj.12303 (2014). 25041354

[b20] Guvenc H. . Impairment of Glioma Stem Cell Survival and Growth by a Novel Inhibitor for Survivin-Ran Protein Complex. Clin. Cancer Res. 19, 631–642, 10.1158/1078-0432.ccr-12-0647 (2013). 23251006 PMC4295559

[b21] Morrison D. J. . Endogenous knockdown of survivin improves chemotherapeutic response in ALL models. Leukemia 26, 271–279, 10.1038/leu.2011.199 (2012). 21844871 PMC3833621

[b22] Song H. . Survivin gene RNA interference inhibits proliferation, induces apoptosis, and enhances radio sensitivity in HeLa cells. Eur. J. Obstet. Gynecol. Reprod. Biol. 136, 83–89, 10.1016/j.ejogrb.2006.07.057 (2008). 17098350

[b23] Singh N., Krishnakumar S., Kanwar R. K., Cheung C. H. A. & Kanwar J. R. Clinical aspects for survivin: a crucial molecule for targeting drug-resistant cancers. Drug Discov. Today 20, 578–587, 10.1016/j.drudis.2014.11.013 (2015). 25433305

[b24] Altieri D. C. Survivin - The inconvenient IAP. Semin. Cell Dev. Biol. 39, 91–96, 10.1016/j.semcdb.2014.12.007 (2015). 25591986 PMC4410054

[b25] Van Impe K. . A nanobody targeting the F-actin capping protein CapG restrains breast cancer metastasis. Breast cancer research: BCR 15, R116, 10.1186/bcr3585 (2013). 24330716 PMC3979033

[b26] Bethuyne J. . A nanobody modulates the p53 transcriptional program without perturbing its functional architecture. Nucleic Acids Res. 42, 12928–12938, 10.1093/nar/gku962 (2014). 25324313 PMC4227789

[b27] Van Overbeke W. . An ER-directed gelsolin nanobody targets the first step in amyloid formation in a gelsolin amyloidosis mouse model. Human molecular genetics 24, 2492–2507, 10.1093/hmg/ddv010 (2015). 25601851

[b28] Van Audenhove I., Debeuf N., Boucherie C. & Gettemans J. Fascin actin bundling controls podosome turnover and disassembly while cortactin is involved in podosome assembly by its SH3 domain in THP-1 macrophages and dendritic cells. Biochimica et biophysica acta 1853, 940–952, 10.1016/j.bbamcr.2015.01.003 (2015). 25601713

[b29] Van Audenhove I. . Fascin Rigidity and L-plastin Flexibility Cooperate in Cancer Cell Invadopodia and Filopodia. The Journal of biological chemistry, 10.1074/jbc.M115.706937 (2016). PMC4861481 26945069

[b30] Muyldermans S. In Annual Review of Biochemistry, Vol. 82 Vol. 82 *Annual Review of Biochemistry* (ed. Kornberg R. D. ) 775–797 (Annual Reviews, 2013).

[b31] Muyldermans S. Single domain camel antibodies: current status. Journal of biotechnology 74, 277–302 (2001). 11526908 10.1016/s1389-0352(01)00021-6

[b32] Delanote V. . An alpaca single-domain antibody blocks filopodia formation by obstructing L-plastin-mediated F-actin bundling. FASEB journal: official publication of the Federation of American Societies for Experimental Biology 24, 105–118, 10.1096/fj.09-134304 (2010). 19726756

[b33] Weaver A. M. . Interaction of cortactin and N-WASp with Arp2/3 complex. Curr. Biol. 12, 1270–1278, 10.1016/s0960-9822(02)01035-7 (2002). 12176354

[b34] Fortugno P. . Regulation of survivin function by Hsp90. Proc. Natl. Acad. Sci. USA 100, 13791–13796, 10.1073/pnas.2434345100 (2003). 14614132 PMC283500

[b35] Henderson B. R. & Eleftheriou A. A comparison of the activity, sequence specificity, and CRM1-dependence of different nuclear export signals. Exp. Cell Res. 256, 213–224, 10.1006/excr.2000.4825 (2000). 10739668

[b36] Zanta M. A., Belguise-Valladier P. & Behr J. P. Gene delivery: A single nuclear localization signal peptide is sufficient to carry DNA to the cell nucleus. Proc. Natl. Acad. Sci. USA. 96, 91–96, 10.1073/pnas.96.1.91 (1999). 9874777 PMC15098

[b37] McBride H. M., Millar D. G., Li J. M. & Shore G. C. A signal-anchor sequence selective for the mitochondrial outer membrane. The Journal of cell biology 119, 1451–1457 (1992). 1334957 10.1083/jcb.119.6.1451 PMC2289765

[b38] John G. B. . The mitochondrial inner membrane protein mitofilin controls cristae morphology. Molecular biology of the cell 16, 1543–1554, 10.1091/mbc.E04-08-0697 (2005). 15647377 PMC551514

[b39] Fransen M., Wylin T., Brees C., Mannaerts G. P. & Van Veldhoven P. P. Human Pex19p binds peroxisomal integral membrane proteins at regions distinct from their sorting sequences. Mol. Cell. Biol. 21, 4413–4424, 10.1128/mcb.21.13.4413-4424.2001 (2001). 11390669 PMC87101

[b40] Van den Abbeele A. . A llama-derived gelsolin single-domain antibody blocks gelsolin-G-actin interaction. Cellular and molecular life sciences: CMLS 67, 1519–1535, 10.1007/s00018-010-0266-1 (2010). 20140750 PMC11115616

[b41] Fortugno P. . Survivin exists in immunochemically distinct subcellular pools and is involved in spindle microtubule function. J. Cell Sci. 115, 575–585 (2002). 11861764 10.1242/jcs.115.3.575

[b42] Van Audenhove I. . Mapping cytoskeletal protein function in cells by means of nanobodies. Cytoskeleton (Hoboken, N.J.) 70, 604–622, 10.1002/cm.21122 (2013). 23818458

[b43] Mikhaylova M. . Resolving bundled microtubules using anti-tubulin nanobodies. Nat. Commun. 6, 10.1038/ncomms8933 (2015). PMC4918323 26260773

[b44] Li F. Z. . Pleiotropic cell-division defects and apoptosis induced by interference with survivin function. Nat. Cell Biol. 1, 461–466, 10.1038/70242 (1999). 10587640

[b45] Maier J., Traenkle B. & Rothbauer U. Real-time analysis of epithelial-mesenchymal transition using fluorescent single-domain antibodies. Sci Rep 5, 10.1038/srep13402 (2015). PMC4544033 26292717

[b46] Rothbauer U. . Targeting and tracing antigens in live cells with fluorescent nanobodies. Nat. Methods 3, 887–889, 10.1038/nmeth953 (2006). 17060912

[b47] Traenkle B. . Monitoring Interactions and Dynamics of Endogenous Beta-catenin With Intracellular Nanobodies in Living Cells. Mol. Cell. Proteomics 14, 707–723, 10.1074/mcp.M114.044016 (2015). 25595278 PMC4349989

[b48] Bourhis E., Hymowitz S. G. & Cochran A. G. The mitotic regulator survivin binds as a monomer to its functional interactor borealin. J. Biol. Chem. 282, 35018–35023, 10.1074/jbc.M706233200 (2007). 17881355

[b49] Presley A. D., Fuller K. M. & Arriaga E. A. MitoTracker Green labeling of mitochondrial proteins and their subsequent analysis by capillary electrophoresis with laser-induced fluorescence detection. J. Chromatogr. B 793, 141–150 10.1016/s1570-0232(03)00371-4 (2003). 12880861

[b50] Otera H. & Fujiki Y. Pex5p Imports Folded Tetrameric Catalase by Interaction with Pex13p. Traffic 13, 1364–1377, 10.1111/j.1600-0854.2012.01391.x (2012). 22747494

[b51] McNew J. A. & Goodman J. M. An Oligomeric Protein is Imported into Peroxisomes *in-vivo*. J. Cell Biol. 127, 1245–1257, 10.1083/jcb.127.5.1245 (1994). 7962087 PMC2120261

[b52] Glover J. R., Andrews D. W. & Rachubinski R. A. Saccharomyces-Cerevisiae Peroxisomal Thiolase is Imported as a Dimer. Proc. Natl. Acad. Sci. USA 91, 10541–10545, 10.1073/pnas.91.22.10541 (1994). 7937990 PMC45057

[b53] Yang X. D., Purdue P. E. & Lazarow P. B. Eci1p uses a PTS1 to enter peroxisomes: either its own or that of a partner, Dci1p. Eur. J. Cell Biol. 80, 126–138, 10.1078/0171-9335-00144 (2001). 11302517

[b54] Islinger M., Li K. W., Seitz J., Volkl A. & Luers G. H. Hitchhiking of Cu/Zn Superoxide Dismutase to Peroxisomes - Evidence for a Natural Piggyback Import Mechanism in Mammals. Traffic 10, 1711–1721, 10.1111/j.1600-0854.2009.00966.x (2009). 19686298

[b55] Freitas M. O. . The peroxisomal protein import machinery displays a preference for monomeric substrates. Open Biol 5, 10.1098/rsob.140236 (2015). PMC4422123 25854684

[b56] Van Audenhove I. . Stratifying fascin and cortactin function in invadopodium formation using inhibitory nanobodies and targeted subcellular delocalization. FASEB journal: official publication of the Federation of American Societies for Experimental Biology 28, 1805–1818, 10.1096/fj.13-242537 (2014). 24414419

[b57] Lossi L., Cocito C., Alasia S. & Merighi A. *Ex vivo* imaging of active caspase 3 by a FRET-based molecular probe demonstrates the cellular dynamics and localization of the protease in cerebellar granule cells and its regulation by the apoptosis-inhibiting protein survivin. Molecular neurodegeneration 11, 34, 10.1186/s13024-016-0101-8 (2016). 27122136 PMC4848850

[b58] Yates J. R., Gilchrist A., Howell K. E. & Bergeron J. J. M. Proteomics of organelles and large cellular structures. Nat. Rev. Mol. Cell Biol. 6, 702–714, 10.1038/nrm1711 (2005). 16231421

[b59] Wiese S. . Proteomics characterization of mouse kidney Peroxisomes by tandem mass spectrometry and protein correlation profiling. Mol. Cell. Proteomics 6, 2045–2057, 10.1074/mcp.M700169-MCP200 (2007). 17768142

[b60] Kikuchi M. . Proteomic analysis of rat liver peroxisome - Presence of peroxisome-specific isozyme of Lon protease. J. Biol. Chem. 279, 421–428, 10.1074/jbc.M305623200 (2004). 14561759

