# Supplementary material for: A new survivin tracer tracks, delocalizes and captures endogenous survivin at different subcellular locations and in distinct organelles
Source: Sci Rep. 2016 Aug 12;6:31177. doi: 10.1038/srep31177 (PMC4981888; doi:10.1038/srep31177)
Supplement: Supplementary Information [file srep31177-s1.pdf]

A new survivin tracer tracks, delocalizes and captures endogenous survivin at different subcellular locations and in distinct organelles.

Authors

Els Beghein[1], Isabel Van Audenhove[1], Olivier Zwaenepoel[1], Adriaan Verhelle[1], Ariane De Ganck[1] and Jan Gettemans*[1]

Affiliations

[1]Department of Biochemistry, Faculty of Medicine and Health Sciences, Campus Rommelaere, A. Baertsoenkaai 3, Ghent University, Ghent, Belgium.

[*]Corresponding author:

Department of Biochemistry,

Faculty of Medicine and Health Sciences,

Campus Rommelaere, Ghent University

Albert Baertsoenkaai 3

B-9000 Ghent, Belgium.

Tel: + 32 9 2649340.

Fax: + 32 9 2649490.

E-mail: jan.gettemans@ugent.be

SUPPLEMENTARY INFORMATION

SUPPLEMENTARY TABLE S1

Tag	Protein of origin	Amino acid sequence	Cellular structure	Localization
NES[4]	Human MAPKK	NLVDLQKKLE ELELDEQQ	Cytoplasm	Cytoplasm
NLS[5]	SV40 Large T antigen	PKKKRKV	Nucleus	Intranuclear
MOM[1]	*Saccharomyces cerevisiae* TOM70	MKSFITRNKTAILAT VAATGTAIGAYYYY	Mitochondria	Mitochondrial outer membrane, outwards
Mitofilin[7]	Human mitofilin	Amino acids 1-199 of human mitofilin	Mitochondria	Mitochondrial inner membrane, towards the intermembrane space
PTS1[8]	Typical peroxisomal matrix proteins	KSKL	Peroxisomes	Peroxisomal matrix

Overview of the different tags used for nanobody and target delocalization. Several tags are used to demonstrate the capacity of the nanobody to delocalize its target outside (NES) or towards (NLS) the nucleus, the mitochondria (MOM and mitofilin) or the peroxisomes (PTS1). The protein of origin is denoted together with the corresponding amino acid sequence of the tag and its presumed cellular localization. NES: nuclear export signal, NLS: nuclear localization signal, MOM: mitochondrial outer membrane, PTS1: type I peroxisomal targeting sequence.

SUPPLEMENTARY FIGURE S1

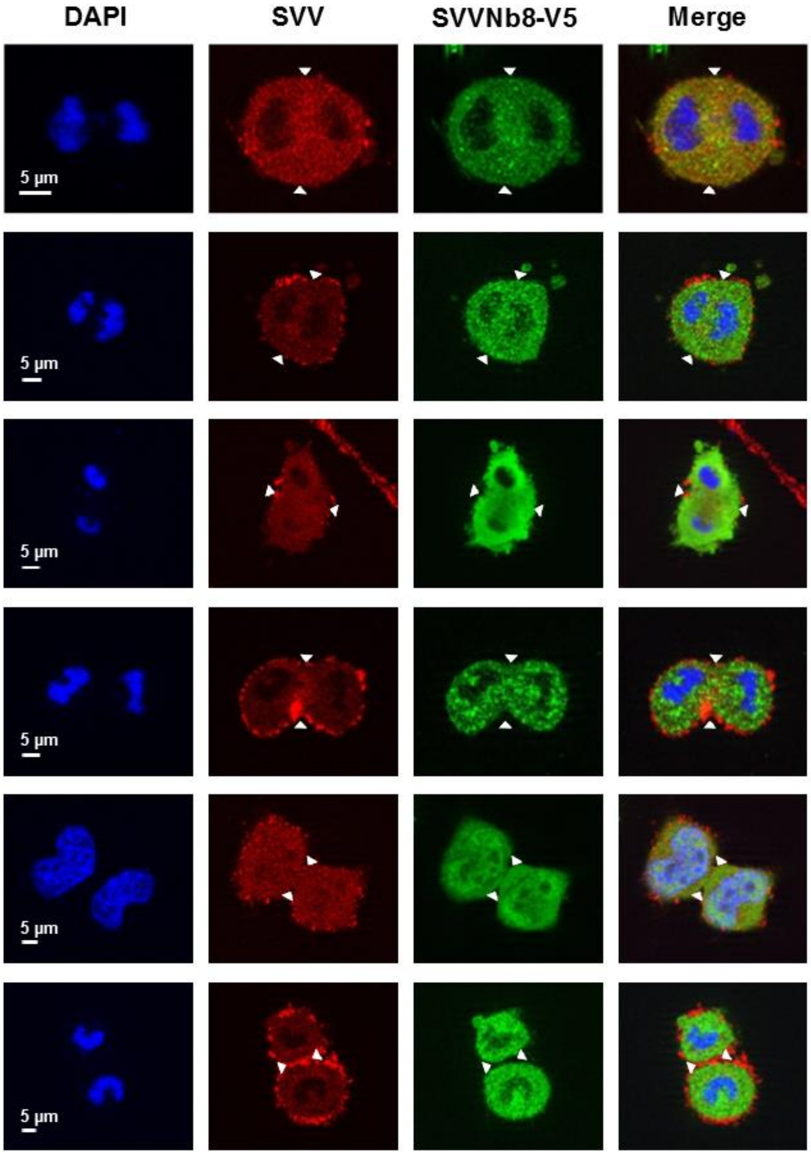

RNAi-mediated SVV depletion abrogates normal SVV and SVVNb8 pattern. Representative epifluorescence images of HeLa cervix cancer cells sequentially transfected with SVV siRNA and SVVNb8 (48 h post-siRNA transfection). Cells were analysed 72 h post-siRNA transfection. SVV is no longer enriched at spindle midzone and midbody, during late anaphase and telophase/cytokinesis, respectively. The same applies to SVVNb8, as no particular enrichment could be observed during different phases of cell division. Arrowheads △ indicate areas of potential cytokinesis, where spindle midzone and midbody formation should be observed in normal dividing cells.

SUPPLEMENTARY FIGURE S2

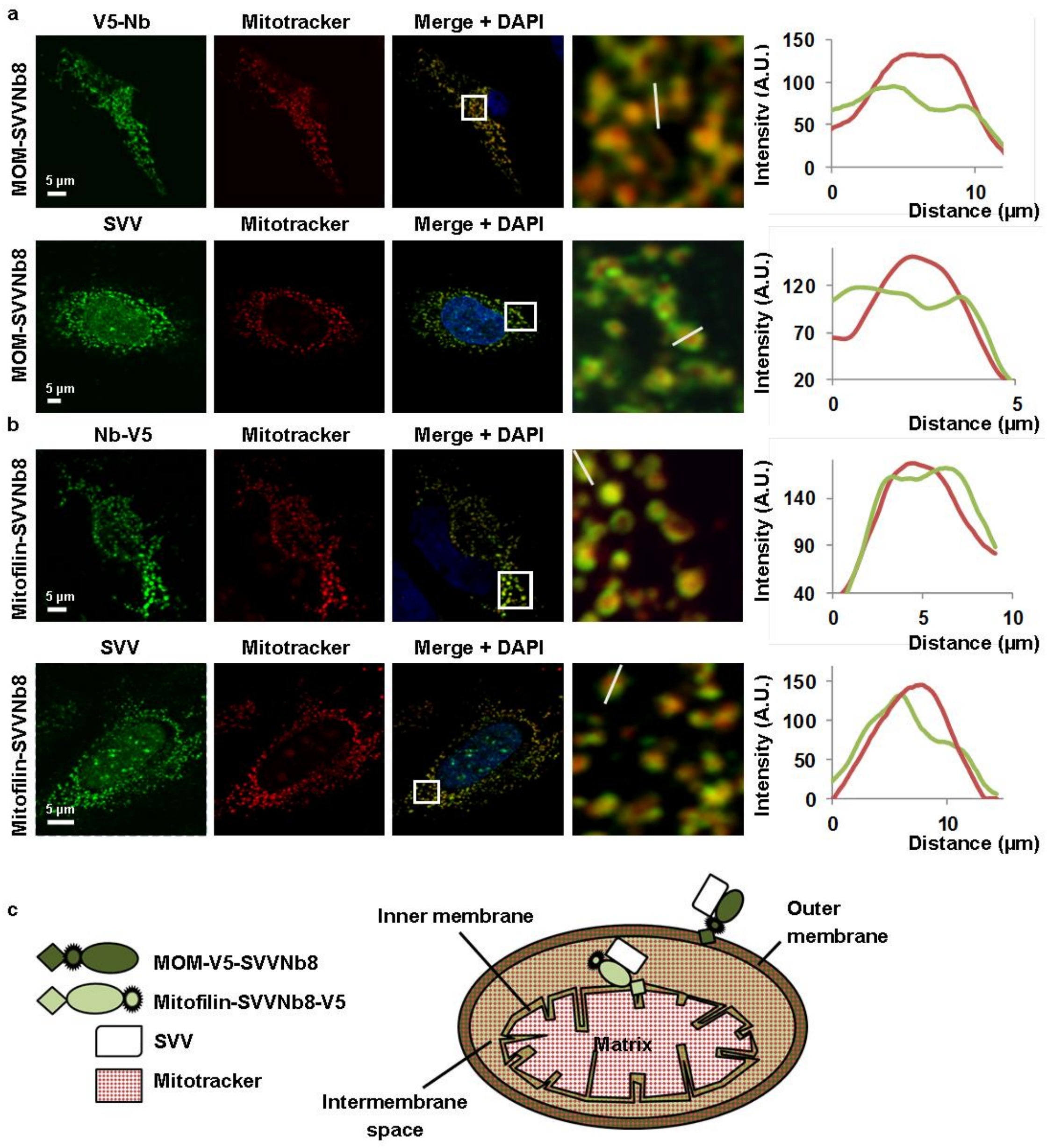

SVVNb8 immobilizes endogenous SVV at the mitochondrial outer rim by means of a MOM or mitofilin tag. HeLa cervix cancer cells were transfected with MOM **(a)** or mitofilin-tagged SVVNb8 **(b)** and imaged with a confocal microscope. As the tags induce anchoring into the outer (MOM) or inner (mitofilin) mitochondrial membrane, the nanobody and SVV are enriched at the border of the mitochondria. Mitotracker labels mitochondria. Nuclei were labelled with DAPI and nanobodies with anti-V5 antibody. Boxed areas are enlarged at the right. Graphs represent corresponding intensity

profiles generated through the white solid lines. **c.** Schematic representation of the different components at the mitochondria. MOM anchors the nanobody and SVV onto the outer membrane (pointing towards the cytoplasm), mitofilin captures the nanobody and SVV at the inner membrane (pointing towards the intermembrane space). Mitotracker covers the outer membrane and the entire inner part of mitochondria, comprising the matrix, inner membrane and intermembrane space. Therefore, nanobody and SVV enrichment occurs at the borders of the Mitotracker labelling.

SUPPLEMENTARY FIGURE S3

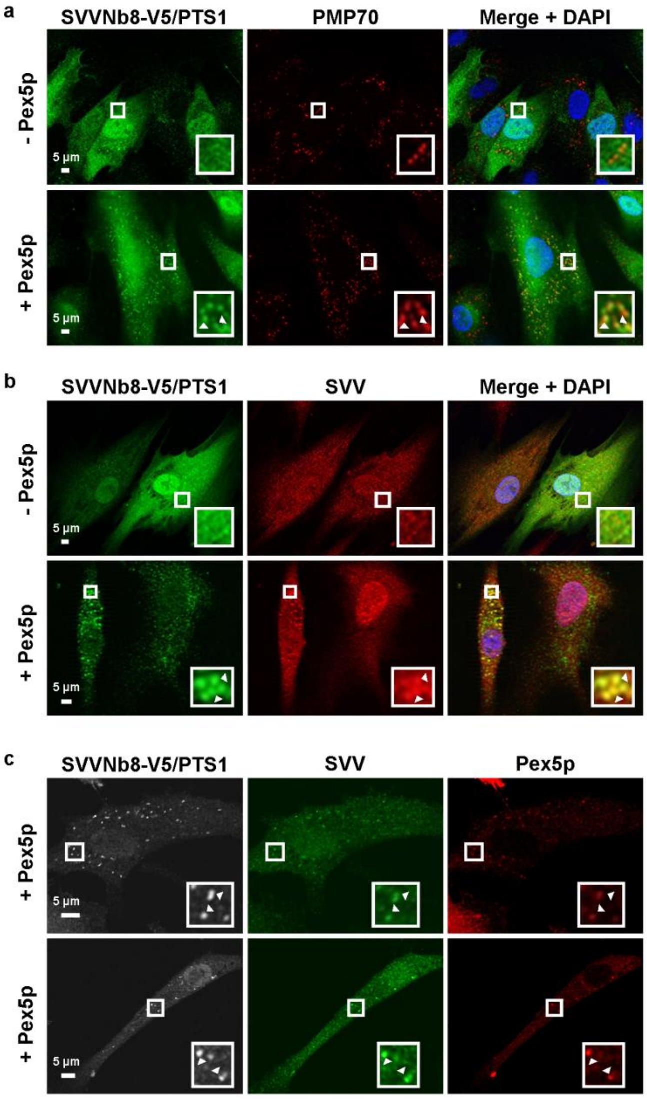

Delocalization of PTS1-tagged nanobody and its interaction partner SVV is Pex5p-dependent. Human Pex5p-deficient fibroblasts were transfected with SVVNb8 only (-Pex5p) or with both SVVNb8 and Pex5p (+ Pex5p). Representative epifluorescence images with PMP70 as a marker of peroxisomes **(a)** or with SVV **(b)** are shown. Only upon Pex5p presence, SVVNb8 is delocalized towards the peroxisomes **(a)** and SVV adopts a peroxisome-like pattern **(b)**. **c.** Representative confocal images revealing colocalization of the nanobody with endogenous SVV and Pex5p upon expression of both SVVNb8 and Pex5p. Nuclei are visualized with DAPI and nanobodies with anti-V5 antibody. Boxed areas are enlarged in the bottom right insets and arrowheads △ indicate colocalization.

SUPPLEMENTARY FIGURE S4

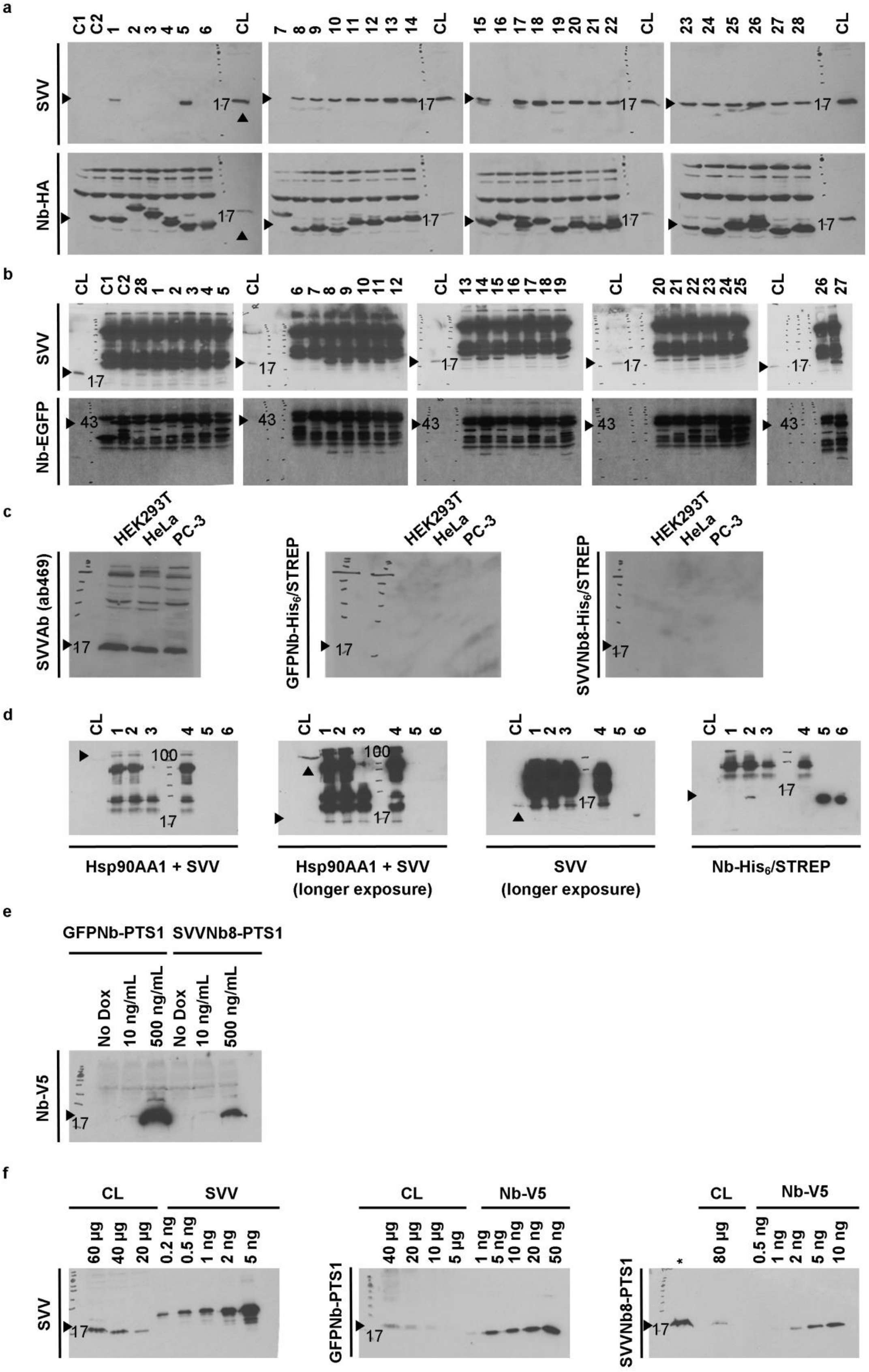

Full Western blots of the experiments shown in **(a)** Figure 1b, **(b)** Figure 1c, **(c)** Figure 2b, **(d)** Figure 3c, **(e)** Figure 7a and **(f)** Fig 7b. Uncropped images are labelled as in the main text and arrowheads △ indicate protein bands of interest. **f.** * depicts 80 μg stable GFP-PTS1 PC-3 crude lysate.

SUPPLEMENTARY METHODS

Generation of survivin (SVV) nanobodies

Nanobodies were obtained in collaboration with the Vlaams Instituut voor Biotechnologie (VIB) Nanobody Service Facility. An alpaca was injected subcutaneously on days 0, 7, 14, 21, 28 and 35 with 100 µg human survivin (SVV; fused to N-terminal His_6-tag) per injection. On day 39, anticoagulated blood was collected for the preparation of lymphocytes. A nanobody library was constructed and screened for the presence of antigen-specific nanobodies. To this end, total RNA from peripheral blood lymphocytes was used as template for first strand cDNA synthesis with oligodT primers. Using this cDNA, the nanobody encoding sequences were amplified by PCR, digested and cloned into the PstI and NotI sites of the phagemid vector pHEN4. In order to isolate SVV nanobodies, several rounds of phage panning were performed on solid-phase coated human His_6-SVV. The enrichment for antigen-specific phages was assessed after each round of panning by comparing the number of phagemid particles eluted from His_6-SVV-coated wells with the number eluted from wells coated with blocking solution only. Individual colonies were analysed by ELISA for the presence of antigen-specific nanobodies in their periplasmic extracts. Finally, positive colonies were analysed by nucleotide sequencing and represented 27 different SVV nanobodies, belonging to 21 different groups. Similarly, immunization of a dromedary with human His_6-SUMO-SVV (VIB Protein Service Facility) resulted in another 28th SVV nanobody.

cDNA cloning

Nanobodies were cloned into pEGFP-N1 (Clontech, Mountain View, CA, USA), pcDNA3.1 V5/His_6 and pcDNA3.1 MOM/V5 (MOM: mitochondrial outer membrane[1]) as described before[2]. The pHEN6c His_6 vector was used as a backbone for the generation of pHEN6c His_6/STREP and pHEN6c V5/SBP/His_6. Insertion of a C-terminal STREP-tag in pHEN6c His_6 was achieved by means of QuickChange Site-Directed Mutagenesis (Stratagene, Agilent Technologies, Santa Clara, CA, USA), using following primers: 5' TGG TCA CAT CCA CAA TTC GAG AAG GAA GCT TCA TAA TAG AAT TCA CTG GCC GT 3' (forward) and 5' TGA AGC TTC CTT CTC GAA TTG TGG ATG TGA

CCA GTG ATG GTG ATG GTG GTG TG 3’ (reverse). A V5-tag was inserted into pHEN6c His_6 as described before[3]. A pUC57 vector containing SBP/His_6 (Custom Gene Synthesis, Eurogentec, Seraing, Belgium) was digested to anneal the SBP-tag into the AgeI/EcoRI sites of the pHEN6c V5/His_6 vector. Nanobodies were subcloned into the pHEN6c His_6/STREP and pHEN6c V5/SBP/His_6 by means of a Cold Fusion Cloning Kit (System Biosciences, Mountain View, CA, USA) using following primers: 5’ CCA GGT GCA GCT GCA GGA GTC TGG GGG AGG ATT 3’ (forward) and 5’ TGA GGA GAC GGT GAC CTG GGT TCC CTG GCC CCA 3’ (reverse). Cloning of a C-terminal nuclear export signal[4] (NES) into the BsrGI-site of pEGFP-N1 (which already contains a nanobody) was performed by means of annealing oligonucleotides : 5’ GTA CAA GGG CGG CGG CGG TTC GGG CGG TAA CCT GGT GGA CCT GCA GAA GAA GCT GGA GGA GCT GGA ACT TGA CGA GCA GCA GCT 3’ (forward) and 5’ GTA CAG CTG CTG CTC GTC AAG TTC CAG CTC CTC CAG CTT CTT CTG CAG GTC CAC CAG GTT ACC GCC CGA ACC GCC GCC GCC CTT 3’ (reverse). A C-terminal nuclear localization signal[5] (NLS) was cloned into pEGFP-N1 as described before[6]. A pUC57 vector containing amino acids 1-199 from human mitofilin[7] (*Q16891*) (Custom Gene Synthesis, Eurogentec) was digested to anneal mitofilin N-terminally from a nanobody into the BamHI/EcoRI sites of the pcDNA3.1 V5/His_6 vector. Insertion of a C-terminal type I peroxisomal targeting sequence PTS1[8] and deletion of the His_6-tag in the pcDNA3.1 V5/His_6 (which already contains a nanobody) was carried out by means of QuickChange Site-Directed Mutagenesis. Following primers were used: 5’ CT ACG CGT ACC GGT AAG AGC AAG CTG TGA CAT CAT CAC CAT CAC 3’ (forward) and 5’ GTG ATG GTG ATG ATG TCA CAG CTT GCT CTT ACC GGT ACG CGT AG 3’ (reverse). Subsequently, nanobody-V5/PTS1 cDNA was subcloned into pLVX-Tight-Puro (Clontech) using: 5’ TGG AGA AGG ATC CGC GGC CGC GCC ACC ATG GCC CAG GTG CAG CTG CAG GAG TCT GGG 3’ (forward) and 5’ CTA CCC GGT AGA ATT CTC ACA GCT TGC TCT TAC C 3’ (reverse) in a Cold Fusion Cloning. Pex5p in pLA140-FLAG vector was a kind gift of Prof. Dr. Marc Fransen (Department of Cellular and Molecular Medicine, KULeuven, Belgium). Throughout this study, a GFP-targeting nanobody was used as a negative control[9]. In summary, following SVV nanobody 8 constructs were used: Nb-His_6/STREP, Nb-

V5/SBP/His_6, Nb-EGFP, Nb-EGFP/NES, Nb-EGFP/NLS, Nb-V5/His_6, MOM/V5-Nb, mitofilin-Nb-V5/His_6 and Nb-V5/PTS1.

Human survivin (SVV or BIRC5, *O15392*; amino acids 2-142) and its BIR (amino acids 15-89), BIR-dimer interfaces (amino acids 2-102) and α-helix (amino acids 100-142) domains were coupled C-terminally of a GST-tag by cloning into pGEX-5X1 (GE Healthcare, Little Chalfont, UK). Following primers were used: 5’ GCC GAA TTC GGT GCC CCG ACG TTG CCC CCT GCC 3’ (forward for full-length SVV and BIR-dimer interfaces domain), 5’ GCC GAA TTC GAA TTT TTG AAA CTG GAC AGA GAA AG 3’ (forward α-helix domain), 5’ GCC GAA TTC AAG GAC CAC CGC ATC TCT ACA TTC 3’ (forward BIR domain), 5’ GGC AGC GGC CGC TCA ATC CAT GGC AGC CAG CTG CTC GAT GGC 3’ (reverse for full-length SVV and α-helix domain), 5’ GGC AGC GGC CGC TCA CAA AAA TTC ACC AAG GGT TAA TTC 3’ (reverse for BIR-dimer interfaces domain), 5’ GGC AGC GGC CGC TCA GAC AGA AAG GAA AGC GCA ACC 3’ (reverse BIR domain). Human cortactin (*Q14247*; amino acids 1-550) was subcloned into pGEX-5X1 using following primers: 5’ GGT CGT GGG ATC CCC GAA TTC ATG TGG AAA GCT TCA GCA GG 3’ (forward) and 5’ T CAG TCA GTC ACG ATG CGG CCG CTG CCG CAG CTC CAC ATA GTT 3’ (reverse) and utilizing the Cold Fusion Cloning Kit. Human SVV (*O15392*; amino acids 1-142) was subcloned into pTrcHis TOPO (Thermo Fisher Scientific) according to the manufacturer’s guidelines in order to attach an N-terminal His_6-tag, using following primers: 5’ AGC ATG GGT GCC CCG ACG TTG CCC CCT GC 3’ (forward) and 5’ TGC GGC CGC TTA ATC CAT GGC AGC CAG CTG CTC GAT GG 3’ (reverse).

Antibodies

Mouse monoclonal anti-HA (12CA5) was purchased from Roche (Basel, Switzerland). Rabbit polyclonal anti-SVV (ab469), rabbit polyclonal anti-PMP70 (ab85550), mouse monoclonal anti-Hsp90AA1 (2G5.G3) and rabbit monoclonal anti-catalase (EP1929Y) were obtained from Abcam (Cambridge, UK). Mouse monoclonal anti-SVV (D-8) was obtained from Santa Cruz Biotechnology (Santa Cruz, CA, USA), rabbit polyclonal anti-EGFP (#2555S) was purchased from Cell Signaling Technology (Danvers, MA, USA), mouse monoclonal anti-His_6 (#631212) was obtained from

Clontech, mouse monoclonal anti-cortactin (4F11) was purchased from Merck Millipore (Darmstadt, Germany) and mouse monoclonal anti-V5 (R960-25) was obtained from Thermo Fisher Scientific (Waltham, MA, USA). Rabbit polyclonal anti-Pex14p was a kind gift of Prof. Dr. Marc Fransen (Department of Cellular and Molecular Medicine, KULeuven, Belgium). Alexa Fluor 488/594 goat anti-mouse or rabbit and Alexa Fluor 647 mouse monoclonal anti-V5 (#451098) were purchased from Thermo Fisher Scientific. ECL anti-mouse or rabbit IgG horseradish peroxidase-linked substrates were purchased from GE Healthcare.

Production and purification of recombinant SVV nanobodies, GST-SVV, GST-SVV fragments, GST-cortactin and His_6-SVV

Recombinant nanobodies in pHEN4, pHEN6c His_6/STREP or pHEN6c V5/SBP/His_6 vector were produced as described before[6]. Briefly, transformed *E. coli* were grown in TB supplemented with 100 µg/ml ampicillin at 37 °C until an OD_{600} of 0.6-0.9 was reached. Nanobody expression was induced by addition of 1 mM IPTG before overnight incubation at 28 °C. Nanobodies were extracted from the periplasm, where they are located due to an N-terminal PelB signal, by means of osmotic burst of the outer membrane using TES buffer (Tris-EDTA-sucrose). Further purification was performed using IMAC and/or gel filtration. For the production of GST constructs in pGEX-5X1, transformed *E. coli* were induced with 1 mM IPTG at OD_{600} of 0.6-0.9 for 3-4 h at 37 °C. Pelleted cells were dissolved in lysis buffer (20 mM Tris pH 8, 150 mM NaCl, 1 mM DTT, 1% Triton X-100, 1 mM PMSF and 1 mM protease inhibitor cocktail) supplemented with 200 µg/ml lysozyme and incubated during 30 min at room temperature. After sonication and pelleting, the GST-fusion proteins were purified from the bacterial lysate by incubation with glutathione sepharose beads (GE Healthcare) for 1-2 h at 4 °C. Beads coated with GST-fusion proteins were used as such for the epitope mapping. For isothermal titration calorimetry, GST-SVV was eluted from the beads with elution buffer (50 mM Tris pH 8, 10 mM DTT, 20 mM glutathione). Recombinant His_6-SVV was produced in *E. coli*, as described for GST-SVV. Cell lysis was performed in 0.5% NP-40 lysis buffer in PBS, with 200 µg/ml lysozyme, 1 mM PMSF and 1 mM protease inhibitor cocktail added. After sonication and pelleting, His_6-tagged SVV was purified using IMAC, followed by ion exchange chromatography and gel filtration.

SVV RNAi

Coverslips were coated with 50 µg/ml rat tail type I collagen (BD Biosciences) in PBS (with Ca^{2+} and Mg^{2+}) for 1 h at 37 °C. HeLa cells were seeded onto the coated coverslips at a density of 60%. 24 h post-seeding, cells were transfected with 55 pmoles validated SVV siRNA (s1458, Thermo Fisher Scientific). 48 h post-siRNA-transfection, cells were transfected with 1 µg SVVNb8-V5/His_6. Transfection of both siRNA and SVVNb8-V5/His_6 was achieved with jetPRIME (Polyplus Transfection Inc.). Immunostaining was performed 24 h later.

SUPPLEMENTARY REFERENCES

1 McBride, H. M., Millar, D. G., Li, J. M. & Shore, G. C. A signal-anchor sequence selective for the mitochondrial outer membrane. *The Journal of cell biology* **119**, 1451-1457 (1992).

2 Delanote, V. *et al.* An alpaca single-domain antibody blocks filopodia formation by obstructing L-plastin-mediated F-actin bundling. *FASEB journal : official publication of the Federation of American Societies for Experimental Biology* **24**, 105-118, doi:10.1096/fj.09-134304 (2010).

3 De Clercq, S. *et al.* Nanobody-induced perturbation of LFA-1/L-plastin phosphorylation impairs MTOC docking, immune synapse formation and T cell activation. *Cellular and molecular life sciences : CMLS* **70**, 909-922, doi:10.1007/s00018-012-1169-0 (2013).

4 Henderson, B. R. & Eleftheriou, A. A comparison of the activity, sequence specificity, and CRM1-dependence of different nuclear export signals. *Exp. Cell Res.* **256**, 213-224, doi:10.1006/excr.2000.4825 (2000).

5 Zanta, M. A., Belguise-Valladier, P. & Behr, J. P. Gene delivery: A single nuclear localization signal peptide is sufficient to carry DNA to the cell nucleus. *Proc. Natl. Acad. Sci. U. S. A.* **96**, 91-96, doi:10.1073/pnas.96.1.91 (1999).

6 Van Audenhove, I. *et al.* Mapping cytoskeletal protein function in cells by means of nanobodies. *Cytoskeleton (Hoboken, N.J.)* **70**, 604-622, doi:10.1002/cm.21122 (2013).

7 John, G. B. *et al.* The mitochondrial inner membrane protein mitofilin controls cristae morphology. *Molecular biology of the cell* **16**, 1543-1554, doi:10.1091/mbc.E04-08-0697 (2005).

8 Fransen, M., Wylin, T., Brees, C., Mannaerts, G. P. & Van Veldhoven, P. P. Human Pex19p binds peroxisomal integral membrane proteins at regions distinct from their sorting sequences. *Mol. Cell. Biol.* **21**, 4413-4424, doi:10.1128/mcb.21.13.4413-4424.2001 (2001).

9 Van den Abbeele, A. *et al.* A llama-derived gelsolin single-domain antibody blocks gelsolin-G-actin interaction. *Cellular and molecular life sciences : CMLS* **67**, 1519-1535, doi:10.1007/s00018-010-0266-1 (2010).
